# Population-level emergence of bedaquiline and clofazimine resistance-associated variants among patients with drug-resistant tuberculosis in southern Africa: a phenotypic and phylogenetic analysis

**DOI:** 10.1016/S2666-5247(20)30031-8

**Published:** 2020-08

**Authors:** Camus Nimmo, James Millard, Lucy van Dorp, Kayleen Brien, Sashen Moodley, Allison Wolf, Alison D Grant, Nesri Padayatchi, Alexander S Pym, François Balloux, Max O'Donnell

**Affiliations:** a Division of Infection and Immunity, University College London, London, UK; b UCL Genetics Institute, University College London, London, UK; c Africa Health Research Institute, Durban, South Africa; d Wellcome Trust Liverpool Glasgow Centre for Global Health Research, Liverpool, UK; e Institute of Infection and Global Health, University of Liverpool, Liverpool, UK; f Department of Medicine, Columbia University Medical Center, New York, NY, USA; g Department of Epidemiology, Columbia University Medical Center, New York, NY, USA; h TB Centre, London School of Hygiene & Tropical Medicine, London, UK; i School of Laboratory Medicine & Medical Sciences, University of KwaZulu-Natal, Durban, South Africa; j CAPRISA-MRC HIV-TB Pathogenesis and Treatment Research Unit, Centre for the Aids Programme of Research in South Africa (CAPRISA), Durban, KwaZulu-Natal, South Africa

## Abstract

**Background:**

Bedaquiline and clofazimine are important drugs in the treatment of drug-resistant tuberculosis and are commonly used across southern Africa, although drug susceptibility testing is not routinely performed. In this study, we did a genotypic and phenotypic analysis of drug-resistant *Mycobacterium tuberculosis* isolates from cohort studies in hospitals in KwaZulu-Natal, South Africa, to identify resistance-associated variants (RAVs) and assess the extent of clofazimine and bedaquiline cross-resistance. We also used a comprehensive dataset of whole-genome sequences to investigate the phylogenetic and geographical distribution of bedaquiline and clofazimine RAVs in southern Africa.

**Methods:**

In this study, we included *M tuberculosis* isolates reported from the PRAXIS study of patients with drug-resistant tuberculosis treated with bedaquiline (King Dinuzulu Hospital, Durban) and three other cohort studies of drug-resistant tuberculosis in other KwaZulu-Natal hospitals, and sequential isolates from six persistently culture-positive patients with extensively drug-resistant tuberculosis at the KwaZulu-Natal provincial referral laboratory. Samples were collected between 2013 and 2019. Microbiological cultures were done as part of all parent studies. We sequenced whole genomes of included isolates and measured bedaquiline and clofazimine minimum inhibitory concentrations (MICs) for isolates identified as carrying any *Rv0678* variant or previously published *atpE, pepQ*, and *Rv1979c* RAVs, which were the subject of the phenotypic study. We combined all whole-genome sequences of *M tuberculosis* obtained in this study with publicly available sequence data from other tuberculosis studies in southern Africa (defined as the countries of the Southern African Development Community), including isolates with *Rv0678* variants identified by screening public genomic databases. We used this extended dataset to reconstruct phylogenetic relationships across lineage 2 and 4 *M tuberculosis* isolates.

**Findings:**

We sequenced the whole genome of 648 isolates from 385 patients with drug-resistant tuberculosis recruited into cohort studies in KwaZulu-Natal, and 28 isolates from six patients from the KwaZulu-Natal referral laboratory. We identified 30 isolates with *Rv0678* RAVs from 16 (4%) of 391 patients. We did not identify any *atpE, pepQ*, or *Rv1979c* RAVs. MICs were measured for 21 isolates with *Rv0678* RAVs. MICs were above the critical concentration for bedaquiline resistance in nine (43%) of 21 isolates, in the intermediate category in nine (43%) isolates, and within the wild-type range in three (14%) isolates. Clofazimine MICs in genetically wild-type isolates ranged from 0·12–0·5 μg/mL, and in isolates with RAVs from 0·25–4·0 μg/mL. Phylogenetic analysis of the extended dataset including *M tuberculosis* isolates from southern Africa resolved multiple emergences of *Rv0678* variants in lineages 2 and 4, documented two likely nosocomial transmission events, and identified the spread of a possibly bedaquiline and clofazimine cross-resistant clone in eSwatini. We also identified four patients with *pepQ* frameshift mutations that may confer resistance.

**Interpretation:**

Bedaquiline and clofazimine cross-resistance in southern Africa is emerging repeatedly, with evidence of onward transmission largely due to *Rv0678* mutations in *M tuberculosis*. Roll-out of bedaquiline and clofazimine treatment in the setting of limited drug susceptibility testing could allow further spread of resistance. Designing strong regimens would help reduce the emergence of resistance. Drug susceptibility testing is required to identify where resistance does emerge.

**Funding:**

Wellcome Trust, National Institute of Allergy and Infectious Diseases and National Center for Advancing Translational Sciences of the National Institutes of Health.

## Introduction

Tuberculosis continues to exert one of the most substantial burdens of infectious disease globally. In 2018, half a million people were diagnosed with tuberculosis resistant to first-line drugs,^1^ and the number of patients receiving treatment for drug-resistant tuberculosis is increasing by over 10% annually.^1^ Bedaquiline, the first new tuberculosis drug in over four decades, has substantially improved drug-resistant tuberculosis outcomes^2^ since it was licensed in 2012. WHO has classified bedaquiline as a group A drug (to be included in all drug-resistant tuberculosis regimens),^3^ and it is central to many clinical trial regimens for drug-susceptible tuberculosis (SimpliciTB, registered with ClinicalTrials.gov, NCT03338621) and tuberculosis resistant to first-line drugs (STREAM2, registered with ClinicalTrials.gov, NCT02409290) and second-line drugs (ZeNix-TB, registered with ClinicalTrials.gov, NCT03086486). Clofazimine was developed for tuberculosis in the 1950s, but given its limited efficacy compared with rifampicin and isoniazid, it has been primarily used to treat leprosy. Clofazimine has been incorporated into WHO guidelines for drug-resistant tuberculosis since 2011,^4^ and is now classified as a group B drug.^3^

Bedaquiline and clofazimine both act by impairing metabolism of mycobacterial energy. Bedaquiline binds to F1F0-ATP synthase preventing proton translocation, while clofazimine acts as a substrate for type II NADH dehydrogenase, the entry point for electrons into the respiratory chain, leading to generation of reactive oxygen species. Bedaquiline resistance is conferred by target mutations in the *atpE* gene,^5^ as well as non-target mutations in *Rv0678* (a negative repressor of the MmpL5 efflux pump)^6^ and *pepQ* (a cytoplasmic peptidase).^7^ Both non-target mutations cause cross-resistance to clofazimine. Clofazimine resistance can also result from mutations in *Rv1979c*, although the mechanism is unclear.^8^ Serial passage of *Mycobacterium tuberculosis* in vitro in the presence of clofazimine selects for development of *Rv0678* variants conferring bedaquiline cross-resistance, suggesting that clinical clofazimine use could lead to bedaquiline resistance even without bedaquiline use.^9^ Identification of resistance is limited by uncertainties over critical concentrations to differentiate resistant and susceptible isolates. The bedaquiline minimum inhibitory concentration (MIC) above which isolates are deemed resistant was set at 0·25 μg/mL on 7H11 agar, although some isolates with *Rv0678* mutations have MICs at or below this concentration.^10^ Because of scarce data, the clofazimine critical concentration has not been established for 7H11 agar, but has been set at 1 μg/mL in mycobacterial growth indicator tube culture.^10^

Bedaquiline clinical trials were first done in KwaZulu-Natal, South Africa, in 2007, and the region became an early adopter with over 3000 patients with drug-resistant tuberculosis receiving bedaquiline between 2012 and 2018 (Iqbal Master, King Dinuzulu Hospital, Durban, personal communication). Likewise, clofazimine has been available in South Africa since 2012. In a clinical report published in February, 2020, that investigated the clinical course and treatment outcomes in a cohort of patients with drug-resistant tuberculosis who were due to start bedaquiline-based therapy in KwaZulu-Natal, we found baseline and emergent *Rv0678* resistance-associated variants (RAVs).^11^ Among sequenced isolates, five (5%) of 92 had baseline *Rv0678* variants. Although none had bedaquiline MICs above the critical concentration, three of five had MICs at the top of the wild-type range and patients with baseline *Rv0678* variants had worse outcomes than those without. Emergent *Rv0678* variants occurred in five (6%) of 87 isolates and were all associated with bedaquiline MICs that were above the critical concentration and worse outcomes. No patient had been previously treated with bedaquiline or clofazimine.

**Research in context**

**Evidence before this study**

Resistance to bedaquiline in clinical isolates of *Mycobacterium tuberculosis* is commonly reported to be caused by mutations in the *Rv0678* gene, with most mutations also conferring cross-resistance to clofazimine. In February 2020, we published a clinical report of baseline and emergent bedaquiline resistance caused by *Rv0678* mutations in patients with drug-resistant tuberculosis in KwaZulu-Natal, South Africa. However, it is also important to know if these mutations caused clofazimine cross-resistance. To establish pre-existing knowledge on the distribution of bedaquiline and clofazimine resistance in southern Africa, we searched PubMed and Google Scholar for articles reporting clinical data in English published since 2000, using the terms “bedaquiline resistance” and “clofazimine resistance” in combination with country names for the Southern African Development Community. We excluded studies that only reported in vitro work. We identified three cohort studies from eSwatini and South Africa reporting a total of 12 isolates with bedaquiline and clofazimine resistance-associated variants and one case report of acquired bedaquiline and clofazimine resistance from South Africa.

**Added value of this study**

We identified 16 patients from our own studies with *Rv0678* mutations (ten of whom were included in the previous clinical report) and report bedaquiline minimum inhibitory concentrations for novel *Rv0678* mutations as well as the range of clofazimine minimum inhibitory concentrations on 7H11 agar. In an extended sequence dataset combining our newly generated whole-genome sequencing data with publicly available sequences from the region, we identified emergence of putative resistance events across southern Africa, two possible cases of nosocomial transmission, and spread of a resistant clone in eSwatini.

**Implications of all the available evidence**

Many different *Rv0678* mutations can cause bedaquiline and clofazimine cross-resistance. Resistance mutations are emerging in multiple locations and there is evidence of spread of resistant strains. This finding is concerning, given the key role bedaquiline, in particular, has in current and future treatment regiments for drug-resistant tuberculosis. Accelerated access to comprehensive drug susceptibility testing in southern Africa and worldwide is urgently required to ensure effective treatment and prevent spread of resistance.

Until early 2020, neither genotypic nor phenotypic drug susceptibility testing for bedaquiline and clofazimine had been routinely performed in KwaZulu-Natal, although phenotypic drug susceptibility testing has now been implemented in South Africa. Reports describing the presence of bedaquiline and clofazimine resistance in southern Africa (defined as the countries of the Southern African Development Community) have been scarce.^12^, ^13^ In this study, we present genotypic and phenotypic data for *M tuberculosis* isolates with RAVs identified from our clinical report^11^ and other unpublished (to date) cohort studies at the Africa Health Research Institute, and report a more extensive microbiological investigation of bedaquiline and clofazimine MICs to investigate the extent of cross-resistance. To better understand the phylogenetic and geographical distribution of bedaquiline and clofazimine RAVs in southern Africa, we then analysed a comprehensive collection of our own and publicly available whole-genome sequencing data.

## Methods

### Study design and isolate selection

In the phenotypic analysis, we included all *M tuberculosis* isolates from patients with *Rv0678* variants in the PRAXIS study (registered with ClinicalTrials.gov, NCT03162107)^11^ and in three other cohort studies of drug-resistant tuberculosis. We also included sequential isolates from one persistently culture-positive patient with extensively drug-resistant tuberculosis with *Rv0678* variants at the KwaZulu-Natal provincial referral laboratory (table 1). Sputum samples for culturing were collected between 2013 and 2019, with most collected from 2016 onwards. Microbiological cultures were done as part of all parent studies. Whole-genome sequencing and measurements of bedaquiline and clofazimine MICs were done as part of this study. RAVs considered able to cause bedaquiline or clofazimine resistance (ie, any *Rv0678* variant and previously published *atpE, pepQ*, and *Rv1979c* RAVs; table 2) were identified from whole-genome sequences.

**Table 1:** List of isolate sources including four cohort studies, a randomised clinical trial, and a provincial referral laboratory

	**Location**	**Design**	**Patients recruited**	**Sputum collection time points**	**Number of patients**	**Number of isolates**
PRAXIS (aim 1), NCT03162107^11^	King Dinuzulu Hospital, Durban	Observational cohort study	HIV co-infected, multidrug-resistant or extensively drug-resistant tuberculosis starting bedaquiline	Baseline, month 1, month 2, month 3, month 4, month 5, month 6, end of treatment	76	145
PRAXIS (aim 2), NCT04032730^11^	King Dinuzulu Hospital, Durban	Randomised controlled trial	HIV co-infected, multidrug-resistant or extensively drug-resistant tuberculosis starting bedaquiline	Baseline, month 2, month 6, end of treatment	41	53
Hlabisa DR-TB, unpublished	Hlabisa Hospital, uMkhanyakude District	Observational cohort study	Multidrug-resistant tuberculosis	Baseline	113	113
CUBS (FIND arm), unpublished	King Dinuzulu Hospital, Durban	Observational cohort study	Multidrug-resistant tuberculosis	Baseline, month 2, month 6, end of treatment	64	81
CUBS (PZAP arm), unpublished	Don Mackenzie Hospital, Durban	Observational cohort study	Multidrug-resistant tuberculosis	Baseline, week 1, week 2, week 4, week 6, month 2, month 3, month 4, month 5, month 6, end of treatment	91	256
Provincial referral laboratory isolates	Inkosi Albert Luthuli Central Hospital, Durban	Ad hoc	Extensively drug-resistant tuberculosis	NA	6	28

ClinicalTrials.gov study registration numbers of isolate sources have been added, if available. All isolates were collected in KwaZulu-Natal, South Africa. A total of 676 isolates were collected from 391 patients. NA=not applicable.

**Table 2:** Mycobacterium tuberculosis variants potentially associated with resistance to bedaquiline or clofazimine

	**Gene**	**Variant**
Bedaquiline	*atpE*	Asp28Ala,^14^ Asp28Gly,^14^ Asp28Asn,^15^, ^16^ Asp28Pro,^16^ Asp28Val,^9^ Leu59Val,^16^ Ala63Pro,^16^ Ala63Val,^15^ Ile66Met^16^
Clofazimine	*Rv1979c*	Val52Gly,^17^ Val351Ala^8^
Bedaquiline and clofazimine	*Rv067*8	Val1Ala (this study),^18^ Val1fs (this study), Val20Gly (this study),^19^ Gln22Pro (this study), Cys46fs (this study),^20^ Asp47fs, Pro48fs,^20^ Glu49fs (this study),^20^ Ala59Val,^21^ Ile67fs (this study), Arg90Cys,^22^ Phe93Ser (this study), Arg94Gln,^5^ Asn98Asp,^22^ Arg109Leu (this study), Ala118Thr (this study), Gly121Arg (this study), Leu136Arg (this study), Met146Thr, ^17^ Glu147fs (this study), Arg156fs (this study)
Bedaquiline and clofazimine	*pepQ*	Ala14fs,^7^ Leu44Pro,^7^ Arg271fs,^7^ any frameshift variant

*Rv0678* variants are listed if associated with intermediate or full resistance to bedaquiline or clofazimine, as reported in previous publications or found in this study (appendix p 7). Previously published resistance-associated variants (RAVs) and any mutation to *Rv0678* were used to select clinical isolates for bedaquiline and clofazimine minimum-inhibitory concentration measurement. All RAVs in this table, including those identified in this study, were used to classify variants identified in our phylogenetic analysis.

We also sequenced the whole genomes of isolates from an unpublished cohort of patients with drug-susceptible tuberculosis at our centre for inclusion in our phylogenetic analysis. All studies and use of provincial referral laboratory isolates (table 1) were approved by the University of KwaZulu-Natal Biomedical Research Ethics Committee.

### Microbiology

In PRAXIS^11^ and three additional cohort studies (table 1), serial sputum samples were obtained at weekly or monthly intervals for mycobacterial growth indicator tube culture and solid culture on Middlebrook 7H11 agar. Provincial referral laboratory isolates were previously cultured in mycobacterial growth indicator tubes and were regrown in Middlebrook 7H9 media from glycerol stocks. Positive cultures from clinical studies underwent phenotypic drug susceptibility testing for first-line and second-line drugs (rifampicin, isoniazid, ethambutol, kanamycin, ofloxacin) on 7H11 agar by the 1% proportion method.

Bedaquiline and clofazimine MICs were measured for all isolates from our clinical studies (table 1) whose genome was identified to carry any *Rv0678* variant or any of the previously published RAVs listed in table 2. When isolates with the same mutation were collected from the same patient at multiple timepoints, MICs were not measured for the isolates derived from samples collected at intermediate timepoints. MICs were measured on 7H11 agar using the proportion method at the Africa Health Research Institute laboratory in Durban, South Africa. Isolates were first grown in 7H9 liquid media adjusted to an optical density at 590 nm of 0·16, from which 10-fold dilutions were prepared. Neat, 10^−2^ and 10^−4^ suspensions were inoculated onto 7H11 agar containing bedaquiline concentrations of 0·03 μg/mL, 0·06 μg/mL, 0·12 μg/mL, 0·25 μg/mL, 0·5 μg/mL, 1·0 μg/mL, 2·0 μg/mL, 4·0 μg/mL, and 8·0 μg/mL, and clofazimine concentrations of 0·06 μg/mL, 0·12 μg/mL, 0·25 μg/mL, 0·5 μg/mL, 1·0 μg/mL, 2·0 μg/mL, and 4·0 μg/mL. *M tuberculosis* strain H37Rv was included in each batch for quality control. All plates were incubated at 37°C and read at 4 and 6 weeks.

### Whole-genome sequencing

DNA was extracted by mechanical ribolysis, which was performed five times at 7000 revolutions per minute for 1 min each (with 1-min intervals on ice) before purification with AMPure XP beads (Beckman Coulter, Indianapolis, IN, USA).^23^ Genomic libraries were prepared with NEBNext Ultra II DNA (New England Biolabs, Ipswich, MA, USA) according to the manufacturer's instructions, and sequenced on a NextSeq 500 (Illumina, San Diego, CA, USA) 300-cycle mid-output run, with 48 samples multiplexed. The whole-genome sequencing bioinformatics pipeline is described in the appendix (p 3).

### Phylogenetic analysis

For our analysis of the phylogenetic and geographical distribution of bedaquiline and clofazimine RAVs in southern Africa, we compiled datasets of lineage 2 and lineage 4 *M tuberculosis* whole-genome sequences obtained from PRAXIS aims 1 and 2, three observational cohort studies, and six patients with persistently culture-positive, extensively drug-resistant tuberculosis from the provincial referral laboratory (table 1). To retrieve further sequences with variants in *Rv0678*, we used BIGSI (version 0.3.8)^24^ to screen publicly available *M tuberculosis* sequences indexed by the National Center for Biotechnology Information (NCBI) Sequencing Read Archive from southern Africa against an internally developed updated index (to April, 2019) for previously reported *Rv0678* RAVs and those we identified in this study (table 2). We also included publicly available sequences from previously published studies in southern Africa. Suitable studies from eSwatini, Malawi, and South Africa were identified as described in the appendix (p 3).^25^, ^26^, ^27^, ^28^, ^29^, ^30^

Sequences were classified by lineage using phylogenetic single-nucleotide polymorphisms (SNPs).^31^ Those containing more than one sublineage-specific SNP were classified as mixed infections and excluded. For lineage 2, H37Rv (GenBank accession NC_000962.3) was included as an outgroup while a lineage 3 outgroup (NCBI Sequencing Read Archive accession SRR1188186) was included for lineage 4. A maximum-likelihood phylogeny was constructed on both sets of SNP alignments using RaxML-NG (version v0.9.0) with 100 bootstrap replicates, a GTR+G substitution model, and performing ascertainment correction for the number of invariant sites. Resulting phylogenies were plotted using ggtree (version 1.16.5).

### Classification of RAVs

Where MICs were not available for public sequence data, *Rv0678* mutations were annotated as resistance-associated if they had previously been associated with a MIC above the critical concentration of 0·25 μg/mL in 7H9 or 7H11 media. To account for the area of technical uncertainty covering strains with MICs equal to the critical concentration of 0·25 μg/mL, we introduced an intermediate category which had previously been proposed to incorporate isolates with a MIC falling outside 95% of wild-type strains.^32^ Strains were labelled susceptible if they were previously associated with a MIC in the wild-type distribution, or unknown if MIC data were not previously reported. The promoter variant −11 C→A was classified as hypersusceptible.^21^ The references used for labelling bedaquiline-resistant strains with *Rv0678* mutations are shown in the appendix (p 7). Variants in *atpE, pepQ*, and *Rv1979c* were considered as potentially causative of resistance if they were located at sites previously associated with resistance (table 2).

### Statistical analysis

A correlation between bedaquiline and clofazimine MICs was calculated with Spearman's correlation using Stata (version 15.1; StataCorp, TX, USA).

### Role of the funding source

The funders of the study had no role in the study design, data collection, data analysis, data interpretation, or writing of the report. All authors had full access to all the data in the study and the corresponding author had final responsibility for the decision to submit for publication.

## Results

We performed whole-genome sequencing on 648 isolates from 385 patients with drug-resistant tuberculosis recruited into cohort studies in KwaZulu-Natal, and 28 isolates from six patients from the KwaZulu-Natal referral laboratory. We identified 30 isolates with *Rv0678* RAVs from 16 (4%) of 391 patients (appendix pp 8–9). RAVs from ten of these patients and associated bedaquiline MICs have been previously reported.^11^ We did not identify any *atpE, pepQ*, or *Rv1979c* variants previously associated with resistance. RAVs were present in pre-treatment samples in seven patients (five previously reported,^11^ two new) and emerged during treatment in eight patients (five previously reported,^11^ three new), with one patient unclassifiable because of the absence of a baseline sample. MICs were measured for 21 isolates with *Rv0678* RAVs and seven isolates with wild-type *Rv0678* (appendix pp 8–9). We did not measure MICs for seven isolates with RAVs as we had measured the MIC for a previous and subsequent sample from the same patient with the same mutations. Additionally, the MICs for the two samples from the provincial referral laboratory were not measured because of equipment unavailability at that location at that time.

Clofazimine MICs for isolates with wild-type *Rv0678* genes ranged from 0·12 to 0·5 μg/mL, while those with *Rv0678* variants ranged from 0·25 to 4·0 μg/mL (table 3). MIC measurement failed for one isolate (P0121 with Phe93Leu RAV), giving MICs for a total of 20 isolates with *Rv0678* RAVs.

**Table 3:** Clofazimine MICs of 20 isolates tested on 7H11 agar with the 1% proportion method, showing number of isolates that have genetically wild-type *Rv0678* and those with *Rv0678* variants

		**0·12 μg/mL**	**0·25 μg/mL**	**0·5 μg/mL**	**1·0 μg/mL**	**2·0 μg/mL**	**4·0 μg/mL**
H37Rv		1	2	..	..	..	..
Clinical isolates							
	Wild type	2	4	1	..	..	..
	Val1Ala/Gly37fs/Arg89Trp	..	..	1	...	..	..
	Val20Gly	..	..	..	..	..	1
	Gln22Pro/Asp47fs	..	..	..	..	2*	..
	Cys46fs	..	..	..	..	1	..
	Cys46fs/Asp47fs	..	..	..	..	2	..
	Asp47fs	..	..	..	..	2	..
	Asp47fs/Ile67fs/Leu136Arg	..	..	..	..	1	..
	Glu49fs	..	1	..	..	1	..
	Ile67fs	..	..	..	..	1	1
	Phe93Ser	..	..	..	2	..	..
	Arg109Leu/Arg156fs	..	..	..	1	1	..
	Ala118Thr	..	..	1	..	..	..
	Glu147fs	..	..	1	..	..	..

*Mycobacterium tuberculosis* strain H37Rv was included in each batch for quality control. WHO has not yet determined a critical concentration for clofazimine on 7H11 agar. MIC measurement failed for one (P0121, carrying the Phe93Leu RAV; appendix p 8, 19) of the 21 clinical isolates with *Rv0678* variants we included in our analysis. MIC=minimum inhibitory concentration.

* One isolate had an additional six *Rv0678* mutations below 15% allele frequency.

MICs were above the critical concentration for bedaquiline resistance in nine (43%) of 21 isolates (table 4; appendix pp 8–9), in the intermediate category in nine (43%) isolates, and within the wild-type range in three (14%) isolates. Isolate P0150 had an Glu49fs mutation at 72% allele frequency and MIC of 0·03 μg/mL that increased by month 2 to 97% allele frequency with MIC of 0·25 μg/mL in the absence of new mutations.^11^ The same results were obtained on repeat testing. Isolate H0147 was phenotypically resistant when tested separately at a binary critical concentration of 0·25 μg/mL, but exhibited slow growth on MIC testing that might have led to a falsely low MIC, highlighting the challenges of MIC reproducibility near critical concentrations. Isolate P0121 had a fixed Phe93Leu mutation.

**Table 4:** Bedaquiline minimum inhibitory concentrations of 21 isolates tested on 7H11 agar with the 1% proportion method, showing number of isolates that have genetically wild-type *Rv0678* and those with *Rv0678* variants

		**<0·03 μg/mL**	**0·03 μg/mL**	**0·06 μg/mL**	**0·12 μg/mL**	**0·25 μg/mL**	**0·5 μg/mL**	**1·0 μg/mL**
H37Rv		..	1	2	..	..	..	..
Clinical isolates								
	Wild type	2	2	3	..	..	..	..
	Val1Ala/Gly37fs/Arg89Trp	..	..	..	..	1	..	..
	Val20Gly	..	..	..	..	1	..	..
	Gln22Pro/Asp47fs	..	..	..	..	..	1*	1
	Cys46fs	..	..	..	..	..	..	1
	Cys46fs/Asp47fs	..	..	..	..	..	..	2
	Asp47fs	..	..	..	..	..	2	..
	Asp47fs/Ile67fs/Leu136Arg	..	..	..	..	..	..	1
	Glu49fs	..	1	..	..	1	..	..
	Ile67fs	..	..	..	..	1	1	..
	Phe93Leu	..	..	..	1	..	..	..
	Phe93Ser	..	..	..	..	2	..	..
	Arg109Leu/Arg156fs	..	..	..	..	2	..	..
	Ala118Thr	..	..	..	1	..	..	..
	Glu147fs	..	..	..	..	1	..	..

*Mycobacterium tuberculosis* strain H37Rv was included in each batch for quality control.

* This isolate had an additional six *Rv0678* mutations below 15% allele frequency.

Distributions of clofazimine and bedaquiline MICs in genetically wild-type and mutated isolates are shown in Table 3, Table 4, indicating an area of technical uncertainty around 0·25–0·5 μg/mL for clofazimine and 0·25 μg/mL for bedaquiline. All clofazimine MICs that were below 0·25 μg/mL occurred in genotypically wild-type isolates, and clofazimine MICs above 0·5 μg/mL all occurred in isolates with *Rv0678* mutations. There was a correlation between bedaquiline and clofazimine MICs (*R*=0·81, p<0·0001; appendix p 4). Three separate variants were identified in *Rv1979c* (appendix pp 8–9): Glu38Asp, Asp286Gly and Arg409Gln. None of the variants in *Rv1979c* had been previously associated with resistance, and all were present in isolates with both low (<0·25 μg/mL) and high (>0·5 μg/mL) clofazimine MICs, suggesting that they were likely to be phylogenetic SNPs.

Of the 29 *Rv0678* RAVs that were identified (between one and eight RAVs per patient), 24 (82%) were heteroresistant at the earliest time point. 23 different amino acid changes were identified, with only three occurring in more than one patient (Asp47fs in four patients, Ile67fs in three patients, and Cys46fs in two patients; appendix pp 8–9). One heterozygous variant increased in frequency to fixation (Arg96Gly in patient L001) while the others remained heterozygous, suggesting clonal interference with other heterozygous *Rv0678* variants in the same sample with coexistence of multiple separate genetic subpopulations.

Of the 16 patients with *Rv0678* variants, eight (50%) were initially infected with lineage 4 *M tuberculosis* strains, seven (44%) with lineage 2 strains, and one (6%) with a lineage 1 strain. These proportions were broadly similar to the frequency of each lineage in our sequenced dataset of drug-resistant tuberculosis from KwaZulu-Natal after excluding mixed infections: 2·8% (10/361) lineage 1, 35·5% (128/361) lineage 2, 1·9% (7/361) lineage 3, and 59·8% (216/361) lineage 4.

We did phylogenetic analyses of *Rv0678* mutations in lineages 2 and 4—the two major *M tuberculosis* lineages present in South Africa and globally. The extended dataset of lineage 2 included 429 isolates (156 newly sequenced from our centre, including 33 from an unpublished drug-susceptible cohort study) listed in the appendix (pp 10–17), and the extended dataset of lineage 4 included 698 isolates (247 newly sequenced from our centre, including 34 from an unpublished drug-susceptible cohort study) listed in the appendix (pp 18–30). In these extended datasets, we found an additional 39 patients with *Rv0678* variants (five in lineage 2 and 34 in lineage 4), and an additional 46 patients with lineage 2 infection who had the −11 C→A promoter mutation associated with low MICs to bedaquiline.^21^ We did not find any *atpE, Rv1979c*, or *pepQ* SNPs associated with resistance. However, we did find four patients in the extended dataset with strains carrying previously unreported *pepQ* frameshift mutations which might cause resistance in the extended dataset: two strains with Asp51fs mutations (at 97·8% and 16·1% allele frequencies) in patients recruited into a study in Cape Town^25^ (NCBI Sequencing Read Archive accessions ERR1873424 and ERR1873561) that were genetically related (one SNP difference), one strain with Ile249fs (53·3% allele frequency) and Ser312fs (43·5% allele frequency) mutations (NCBI Sequencing Read Archive accession SRR1175307), and one patient with a Val273fs (12·1% allele frequency) mutation in combination with an *Rv0678* G121R mutation (NCBI Sequencing Read Archive accession SRR1167167). All four strains were multidrug-resistant or extensively drug-resistant tuberculosis as determined by whole-genome sequencing.

The phylogenetic relatedness of the isolates with *Rv0678* variants likely to be associated with resistance is shown in the figure and the appendix (pp 5–6). In the extended dataset of lineage 2 (our own plus publicly available data), *Rv0678* RAVs were identified in 20 sequences from 18 patients. Apart from three sequential samples from patient P0082 and the previously described possible nosocomial transmission to patient P0101 at King Dinuzulu Hospital (figure),^11^ the isolates from the other 16 patients contained eight different mutations and were distributed throughout the phylogeny, indicating multiple independent emergence events. Five patients were from cohort studies at the Africa Health Research Institute, two were from a study in KwaZulu-Natal,^26^ and three from studies in Western Cape.^25^, ^27^

**Figure:**
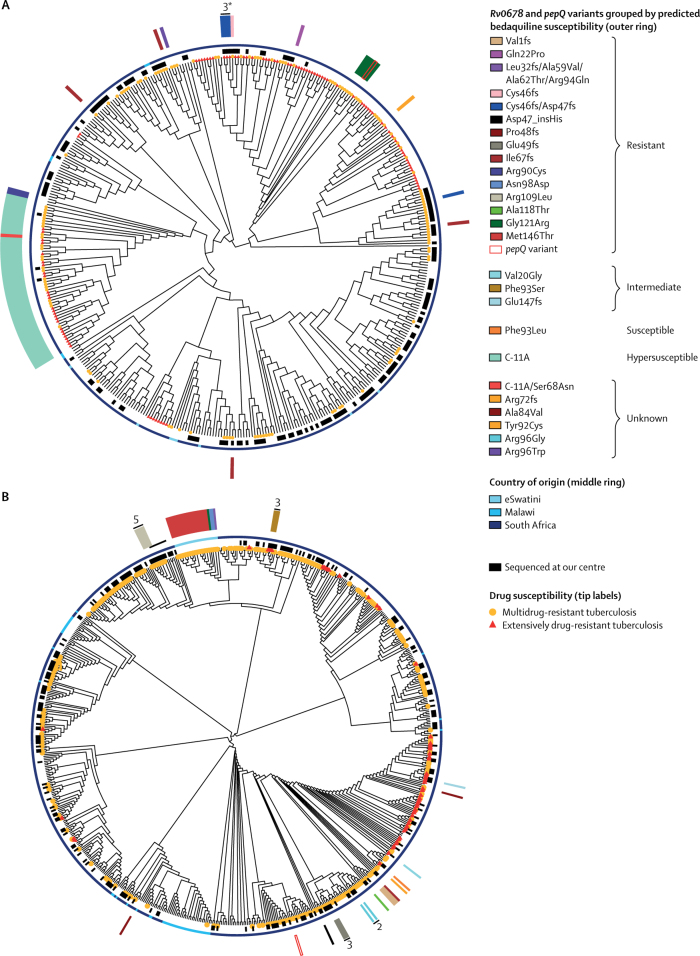
Phylogeny of southern African lineage 2 (A) and lineage 4 (B) *Mycobacterium tuberculosis* strains showing bedaquiline resistance profiles Where multiple isolates originate from the same patient, this is indicated with a black line and the number of isolates. *Indicates likely nosocomial transmission (see main text).

Among the lineage 4 strains, we identified a clade of 20 isolates from patients in eSwatini with a Met146Thr mutation,^33^ although both resistant and susceptible bedaquiline MICs have previously been reported with this variant.^17^, ^22^ Overall *Rv0678* variants were identified in 49 isolates from 40 patients in the extended lineage 4 dataset, of which six sequences from separate patients were identified from a previous study in KwaZulu-Natal.^26^ Genetically identical sequences were isolated from three patients in Manguzi Hospital in KwaZulu-Natal within one month of each other (NCBI Sequencing Read Archive accessions SRR1184367, SRR1184369, and SRR1180407) that shared a Val1fs mutation, while the other samples all appeared to represent independent resistance emergence events.

## Discussion

We identified a wide diversity of *Rv0678* mutations in the genomes of clinical isolates from hospitals in KwaZulu-Natal that we sequenced and of publicly available strains in southern Africa, many of which have not been reported previously. This finding probably reflects the fact that *Rv0678* is non-essential, and since the underlying resistance mechanism causing bedaquiline and clofazimine cross-resistance is loss of function (similar to pyrazinamide resistance caused by *pncA* mutations), there are potentially a large number of resistance-conferring variants. Most mutations with an associated MIC were likely to cause intermediate or full bedaquiline resistance. Initial reports of bedaquiline MICs in patients with *Rv0678* mutations suggested a roughly even split of susceptible, intermediate, and resistant MICs,^5^ whereas here the majority of mutations seemed to have potential clinical significance. In our previous study,^11^ we identified worse clinical outcomes among the five patients with baseline *Rv0678* variants even though MICs were susceptible or intermediate.^11^ Furthermore, the range of different mutations we report in this study, spanning the length of the *Rv0678* gene, means that development of a rapid molecular diagnostic could be challenging (as has been the case for pyrazinamide) and targeted or whole-genome sequencing is likely to provide the best solution to identify *Rv0678* RAVs.

We did not identify any previously reported *atpE* RAVs, which have been infrequently identified in clinical samples. *atpE* mutations have been previously found in vitro, but might have too high a fitness cost to occur frequently in clinical samples. Although we did not find any *pepQ* mutations in our own dataset, we did identify in publicly available data four strains with *pepQ* frameshift mutations that could confer resistance. However, while two previously reported *pepQ* frameshift variants conferred resistance,^7^ this has not been tested for other variants and therefore these findings should be interpreted with caution. Nevertheless, presence of these mutations suggests that it will be important to implement ongoing monitoring for *pepQ* variants with phenotypic testing.

We did not find evidence of *Rv1979c* mutations causing clofazimine resistance, with the mutations that we identified most likely to be phylogenetic variants. In the extended dataset, we found no previously reported *Rv1979c* variants associated with resistance, but this search is likely limited by the small number of reported mutations with correlated phenotypic data. Further work is needed to discern a critical concentration for clofazimine on 7H11 agar, although our data suggest that both wild-type and mutant MICs cluster around the proposed critical concentration, challenging the establishment of a single meaningful cut-off, as is the case for bedaquiline.

Most *Rv0678* variants were heterozygous and did not reach fixation. Four patients had *M tuberculosis* containing more than one heterozygous variant coexisting simultaneously. This points to multiple emergences of resistance within each patient, which was most extreme for one patient in particular who had eight different heterozygous variants by month 6. Heteroresistance has been identified with other tuberculosis drugs (although less frequently) and is most common for fluoroquinolones, where approximately 20% of resistance could be conferred by heterozygous RAVs.^34^ Importantly, heteroresistance might confound MIC testing if drug-susceptible populations predominate in the phenotypic drug-susceptibility testing media, hiding the true dynamics of *M tuberculosis* in the host. An example from this study is isolate P0150, where the Glu49fs mutation (which has previously been associated with bedaquiline resistance)^20^, ^32^ was present at 72% allele frequency at baseline with a bedaquiline MIC of 0·03 μg/mL, and increased by month 2 to 97% allele frequency with an MIC of 0·25 μg/mL in the absence of new mutations.^11^

The phylogenies we constructed for the two major epidemic *M tuberculosis* lineages in southern Africa (lineages 2 and 4) show RAVs across both lineages and in genetically distant strains, indicating multiple resistance emergence events. We additionally identified strains from earlier studies in KwaZulu-Natal carrying previously unidentified bedaquiline resistance. Worryingly, in addition to the possible nosocomial transmission events at King Dinuzulu Hospital^11^ and Manguzi Hospitals (three patients sharing a strain with the Val1fs mutation), there is evidence of spread of a clone (genetically similar strains from different patients) carrying the Mer146Thr mutation in eSwatini which might confer raised bedaquiline MICs and requires further characterisation.^17^, ^22^

These isolates were cultured from samples collected between 2009 and 2012, before the introduction of bedaquiline or clofazimine to eSwatini's national tuberculosis programme, suggesting that the mutation was selected for by another evolutionary pressure. For example, it could have originated in a patient treated with clofazimine for leprosy or an azole for fungal disease who was co-infected with *M tuberculosis*, with the azole for fungal disease a more likely possibility given the low incidence of leprosy in eSwatini and the high incidence of HIV and potential for opportunistic fungal infections.^6^, ^35^, ^36^ Given that bedaquiline and clofazimine have both been used in eSwatini since 2014–15, urgent action is required to ensure that unidentified bedaquiline and clofazimine resistance does not spread in a similar fashion to that of rifampicin resistance conferred by the *rpoB* Ile491Phe mutation and missed by Xpert MTB/RIF.^33^ The significance, if any, of the −11 C→A promoter mutation remains uncertain. Given its phylogenetic restriction to one clade of lineage 2, it might represent a chance mutation or adaptation to an as yet unidentified pressure.

One limitation of this study is that we only measured bedaquiline and clofazimine MICs on isolates from our centre with RAVs, because of the time and cost constraints. The resistance mechanisms mediated by *Rv1979c* and *pepQ* remain largely unexplained, so we will have missed unreported RAVs in these genes and in other, as yet unidentified genes. In the extended phylogenetic dataset we used a comprehensive approach to identify all *Rv0678* RAVs, but were unable to use this approach for other resistance-associated genes because of the greater presence of phylogenetic SNPs and lack of phenotypic correlation in other genes. We will therefore not have identified resistance conferred by as yet unidentified mechanisms. Previously reported resistance associated with *Rv0678* variants was based on published reports and our own data, but for many RAVs only small numbers of reports exist, making it challenging to establish a robust causative relationship. Both data from our centre and publicly uploaded datasets are incomplete and likely to both miss resistance that is present and under-represent the large number of fully drug-susceptible strains circulating. Among publicly uploaded data, we aimed to exclude duplicate samples by ensuring samples had unique BioSample identifiers and patient identification numbers. However, it is not possible to completely exclude that some isolates were sequenced more than once and uploaded with different identifiers.

Our data suggest that bedaquiline and clofazimine cross-resistance mediated by *Rv0678* mutations is emerging throughout southern Africa. The rollout of bedaquiline and clofazimine treatment, in the setting of limited drug-susceptibility testing and inadequate adherence support, could allow the spread of emergent resistance. Designing strong regimens to be used programmatically such as those currently in use in South Africa, which include highly effective drugs such as linezolid, might help reduce emergence of resistance. Additionally, drug susceptibility testing is required to identify regions where resistance does emerge, which could involve a combination of genotypic and phenotypic methods.

## Data sharing

Raw sequencing reads are uploaded onto NCBI Sequencing Read Archive under BioProject PRJNA559528.
