# Supplementary material for: Population-level emergence of bedaquiline and clofazimine resistance-associated variants among patients with drug-resistant tuberculosis in southern Africa: a phenotypic and phylogenetic analysis
Source: Lancet Microbe. 2020 Aug;1(4):e165–74. doi: 10.1016/S2666-5247(20)30031-8 (PMC7416634; doi:10.1016/S2666-5247(20)30031-8)
Supplement: Supplementary appendix [file mmc1.pdf]

THE LANCET
Microbe

Supplementary appendix

This appendix formed part of the original submission and has been peer reviewed. We post it as supplied by the authors.

Supplement to: Nimmo C, Millard J, van Dorp L, et al. Population-level emergence of bedaquiline and clofazimine resistance-associated variants among patients with drug-resistant tuberculosis in southern Africa: a phenotypic and phylogenetic analysis. *Lancet Microbe* 2020; **1:** e165–74.

RESEARCH IN CONTEXT 2

SUPPLEMENTARY METHODS 3

SUPPLEMENTARY FIGURES 4

SUPPLEMENTARY TABLES 7

ADDITIONAL REFERENCES 31

Research in Context

The following studies were identified during a search of PubMed and Google Scholar for articles reporting clinical data in English published since 2000 using the terms “bedaquiline resistance” and “clofazimine resistance” in combination with country names for the Southern African Development Community conducted on 13th May 2020. Studies reporting only *in vitro* results and review articles were excluded.

Dheda K, Limberis JD, Pietersen E, Phelan J, Esmail A, Lesosky M, et al. Outcomes, infectiousness, and transmission dynamics of patients with extensively drug-resistant tuberculosis and home-discharged patients with programmatically incurable tuberculosis: a prospective cohort study. Lancet Respir Med. 2017 Apr;5(4):269-281.

Makhado NA, Matabane E, Faccin M, Pinçon C, Jouet A, Boutachkourt F, et al. Outbreak of multidrug-resistant tuberculosis in South Africa undetected by WHO-endorsed commercial tests: an observational study. Lancet Infect Dis. 2018;18(12):1350–9.

de Vos M, Ley SD, Wiggins KB, Derendinger B, Dippenaar A, Grobbelaar M, et al. Bedaquiline Microheteroresistance after Cessation of Tuberculosis Treatment. N Engl J Med. 2019 May 30;380(22):2178-2180.

Klopper M, Heupink TH, Hill-Cawthorne G, Streicher EM, Dippenaar A, De Vos M, et al. A landscape of genomic alterations at the root of a near-untreatable tuberculosis epidemic. BMC Med. 2020;18(1).

Supplementary Methods

Whole genome sequencing bioinformatics pipeline

Adapters were removed using Trim Galore v0.3.7. Reads were mapped to H37Rv (NC_000962.3) with BBMap v38.32 specifying a 98% identity threshold, de-duplicated with Picard Tools v2.20 and then mean genome coverage measured with Qualimap v2.21. FreeBayes v1.2 was used to call variants with supported by ≥4 reads, including ≥1 on each strand, with mapping quality ≥20 and base quality ≥30. Functional consequences of the identified variants were annotated with snpEff.

Additional studies included in phylogenies

A selection of additional whole genome sequences from studies in southern Africa were included to provide phylogenetic context. Where isolates with *Rv0678* resistance-associated variants had been identified by BIGSI or PYGSI from a published study, other whole genome sequences from that study were also included.

Additional lineage 2 genomes from southern Africa were identified by screening the National Center for Biotechnology Information (NBCI) Sequencing Read Archive with BIGSI for *Mycobacterium tuberculosis* whole genome sequences collected from Southern African Development Community countries with the phylogenetically definitive nucleotide change in *rpsA* 636 A→C.(1)

Additional lineage 4 genomes from Southern African Development Community countries were included from a comprehensive global study of lineage 4.(2)

Supplementary Figures

Supplementary Figure 1. Correlation between bedaquiline and clofazimine MICs in isolates with a wild-type (red squares) and mutated (blue circles) *Rv0678* genes.

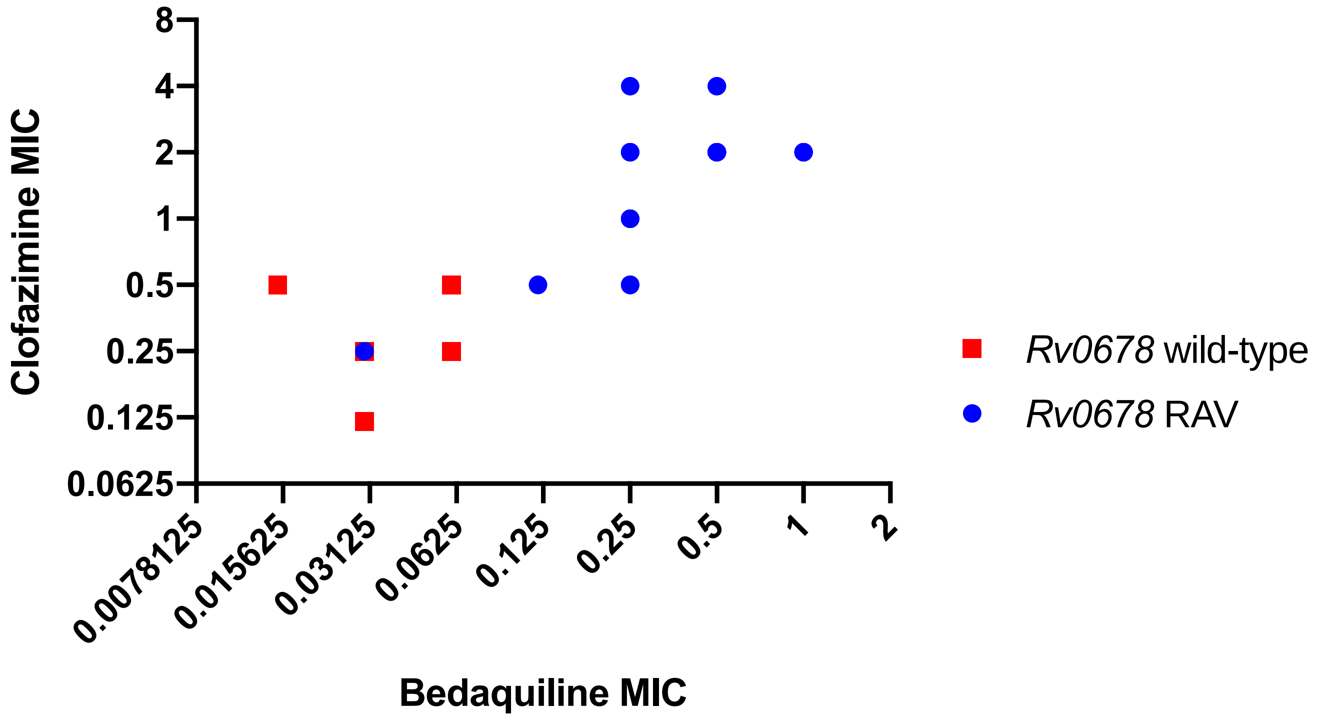

Supplementary Figure 2 (page 5). Linear phylogenetic tree of lineage 2 sequences, indicating study identification number (format XX_X0000X) for samples sequenced for this study, or NCBI Sequencing Read Archive / European Nucleotide Archive accession number for publicly available samples (starting SRR/ERR). Annotations indicate resistance-associated variants and previously observed resistance profile associated with the variant.

Supplementary Figure 3 (page 6). Linear phylogenetic tree of lineage 4 sequences, indicating study identification number (format XX_X0000X) for samples sequenced for this study, or NCBI Sequencing Read Archive / European Nucleotide Archive accession number for publicly available samples (starting SRR/ERR). Annotations indicate resistance-associated variants and previously observed resistance profile associated with the variant.

Supplementary Figure 2

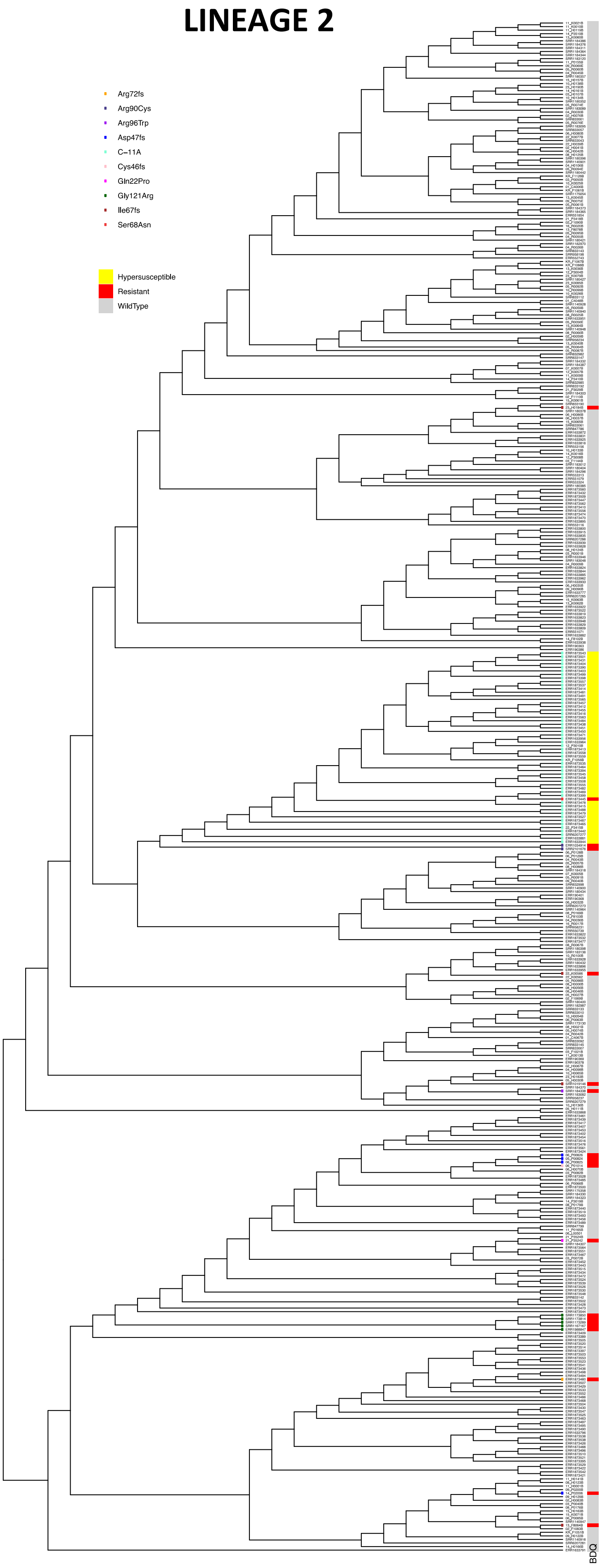

Supplementary Figure 3

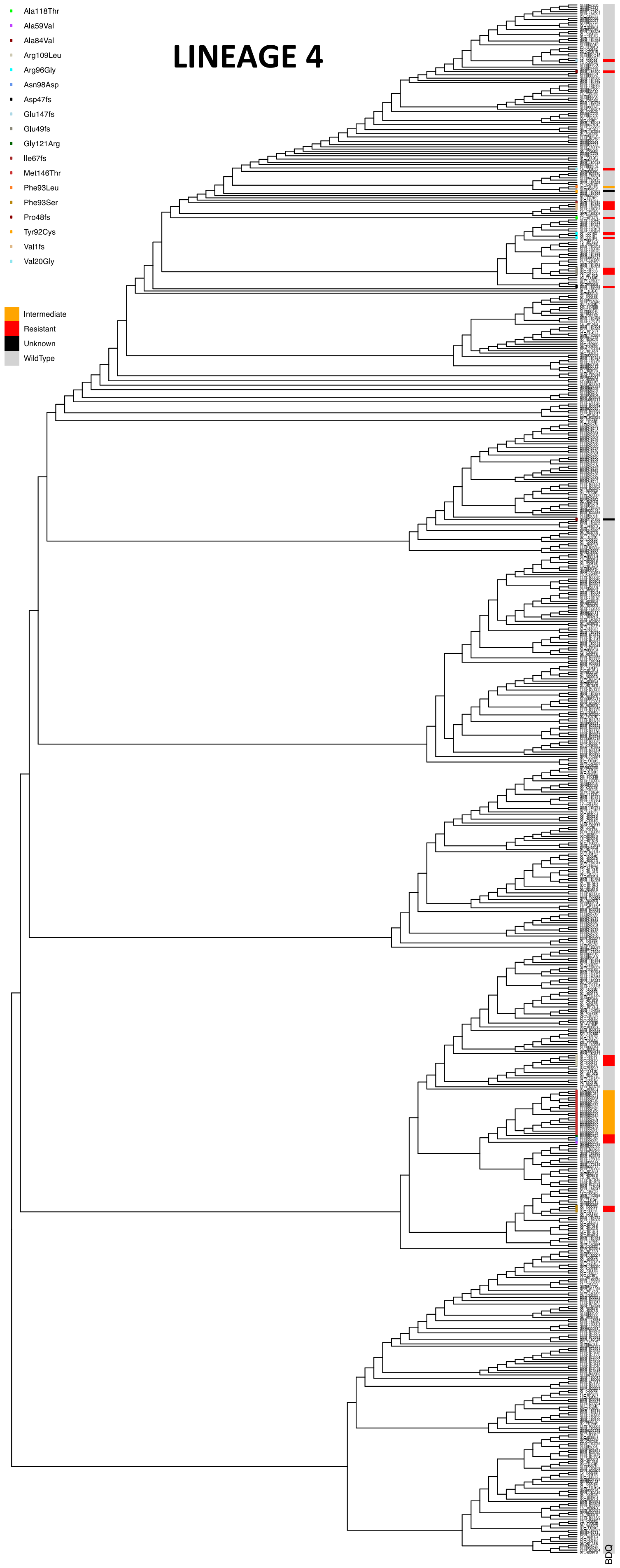

Supplementary Tables

Supplementary Table 1. Previously observed bedaquiline resistance associated with *Rv0678* variants included in the extended datasets used for phylogenetic reconstruction. int = associated with intermediate MIC, comb = only seen in combination with other *Rv0678* variants.

**Observed resistance**	**Variant**	**Associated with MIC**	
		**Resistant / intermediate**	**Susceptible**
Resistant	Val1Ala	Somoskovi (3), this study	
	Gln22Pro	This study	
	Ala59Val	Villellas (4)	
	Arg90Cys	Yang (5)	
	Arg94Gln	Andries (6)	
	Asn98Asp	Yang (5)	
	Ala118Thr	This study	
	Leu136Arg	This study	
	Met146Thr	Xu (7)	Yang (5)
	Frameshift variants<br>Val1fs<br>Cys46fs<br>Asp47fs<br>Pro48fs<br>Glu49fs<br>Ile67fs<br>Arg156fs	 This study<br>This study, Andres (8)<br>Andres (8)<br>Andres (8)<br>This study (int), Andres (8)<br>This study<br>This study	
Intermediate	Val20Gly	This study, Ghodousi (9)	
	Phe93Ser	This study	
	Arg109Leu	This study	
	Gly121Arg	This study	
	Frameshift variants<br>Glu147fs	 This study	
Susceptible	Phe93Leu		This study
Hypersusceptible	C-11A		Andries (6)
Unknown	Ala62Thr		
	Ser68Asn		
	Ala84Val		
	Tyr92Cys		
	Arg96Gly		
	Arg96Thr		
	Frameshift variants<br>Arg72fs		

Supplementary Table 2. Isolates from our centre with *Rv0678* variants, and preceding isolates from the same patient if available. Patients previously reported in Nimmo et al (10) are denoted with the corresponding patient identifier in brackets. All variants in *Rv0678* and *Rv1979c* are shown. No resistance-associated variants were found in *pepQ* or *atpE*. Drug resistance classification was determined by phenotypic testing. Bedaquiline and clofazimine MICs are shown where tested. Patients are referred to by five-character study identifiers. *There was discordance with the same sample tested separately at a critical concentration of 0.25μg/mL appearing phenotypically resistant.

Study ID (Key to Nimmo et al) (10)	Time-point	Sequencing ID	Rv0678 variant (frequency)	Variants in Rv1979c	Drug resistance classification (phenotypic DST)	Bedaquiline MIC	Clofazimine MIC
P0027 (A)	Baseline	06_P0027B	Arg109Leu (67.9%)<br>Arg156fs (25.8%)		Pre-XDR (F)	0.25	1.0
	Month 1	01_P00271	Arg109Leu (86.8%)<br>Arg156fs 16.5%)				
	Month 3	01_P00273	Arg109Leu (86.7%)<br>Arg156fs (9.5%)				
	Month 4	01_P00274	Arg109Leu (29.1%)<br>Arg156fs (69.6%)				
	Month 5	07_P00275	Arg109Leu (27.8%)<br>Arg156fs (69.2%)				
	Month 6	04_P00276	Arg109Leu (23.9%)<br>Arg156fs (57.1%)		Pre-XDR (F)	0.25	2.0
P0150 (B)	Baseline	08_P0150B	Glu49fs (72.0%)	Rv1979c Glu38Asp (100.0%)	Pre-XDR (F)	0.03	0.25
	Month 1	08_P01501	Glu49fs (94.2%)	Rv1979c Glu38Asp (100.0%)			
	Month 2	08_P01502	Glu49fs (96.7%)	Rv1979c Glu38Asp (100.0%)		0.25	2.0
P3016 (C)	Baseline	14_P3016B	Val20Gly (100.0%)	Rv1979c Glu38Asp (100.0%)	Pre-XDR (SLI)	0.25	4.0
P0005 (D)	Baseline	03_P0005B	Phe93Ser (100.0%)		Pre-XDR (F)	0.25	1.0
	Month 1	09_P00051	Phe93Ser (100.0%)				
	Month 5	06_P00055	Phe93Ser (100.0%)				
	Month 6	03_P00056	Phe93Ser (100.0%)			0.25	1.0
P0121 (E)	Baseline	04_P0121B	Phe93Leu (100.0%)	Rv1979c Glu38Asp (100.0%)	XDR	0.12	Failed x3

P0082 (F)	Baseline	03_P0082B	Wild type	Rv1979c Arg409Gln (100.0%)	XDR	0.06	0.25
	Month 4	05_P00824	Asp47fs (93.4%)	Rv1979c Arg409Gln (100.0%)		0.5	2.0
	Month 5	06_P00825	Cys46fs (35.9%) Asp47fs (53.3%)	Rv1979c Arg409Gln (100.0%)		1.0	2.0
	Month 6	06_P00826	Cys46fs (23.1%) Asp47fs (69.8%)	Rv1979c Arg409Gln (100.0%)	XDR	1.0	2.0
P0101 (G)	Baseline	04_P0101B	Wild type	Rv1979c Glu38Asp (100.0%)	XDR	0.03	0.12
	Month 4	06_P01014	Cys46fs (97.7%)	Rv1979c Arg409Gln (100.0%)		1.0	2.0
P0200 (H)	Baseline	09_P0200B	Wild type	Rv1979c Arg409Gln (100.0%)	XDR	0.06	0.25
	Month 6	14_P02006	Asp47fs (92.3%)	Rv1979c Arg409Gln (100.0%)	XDR	0.5	2.0
P3505 (I)	Baseline	13_P3505B	Wild type	Rv1979c Glu38Asp (100.0%)	XDR	0.03	0.25
	Month 6	25_P35056	Glu147fs (100.0%)	Rv1979c Glu38Asp (100.0%)		0.25	0.5
P3524 (J)	Baseline	21_P3524B	Wild type	Rv1979c Arg409Gln (100.0%)	XDR	<0.03	0.25
	Month 2	21_P35242	Gln22Pro (85.1%) Asp47fs (12.6%)	Rv1979c Arg409Gln (100.0%)		1.0	2.0
	Month 6	25_P35246	Glu22Pro (9.8%) Asp47fs (45.2%) Ala57Glu (10.6%) Arg72Thr (9.2%) Asp88fs (12.0%) Asp88Ala (13.0%) Glu121Arg (6.0%) Leu122Pro (7.4%)	Rv1979c Arg409Gln (100.0%)	XDR	0.5	2.0
L001	Baseline	06_L00101	Wild type	Rv1979c Arg409Gln (100.0%)			
	Month 12	07_L00102	Arg96Gly (8.8%)	Rv1979c Arg409Gln (100.0%)			
	Month 19	06_L00103	Arg96Gly (100.0%)	Rv1979c Arg409Gln (100.0%)			
H0147	Baseline	12_H0147B	Ala118Thr (100.0%)	Rv1979c Glu38Asp (100.0%)	MDR	0.12*	0.5
H0184	Baseline	23_H0184B	Ile67fs (8.3%)		MDR	0.25	2.0
F8064	Month 20	13_F8064B	Asp47fs (7.1%) Ile67fs (54.3%) Leu136Arg (22.6%)	Rv1979c Arg409Gln (100.0%)	Pre-XDR	1.0	2.0
K0056	Week 2	22_K00562	Wild type		Rifampicin monoresistant	0.06	0.5
	Week 6	22_K00566	Ile67fs (95.1%)			0.5	4.0
K0084	Baseline	24_K0084B	Wild type	Rv1979c Asp286Gly (100.0%)		<0.03	0.5
	Week 2	24_K00842	Wild type	Rv1979c Asp286Gly (100.0%)			
	Month 6	26_K0084K	Val1Ala (62.8%) Gly37fs (8.1%) Arg89Trp (11.8%)	Rv1979c Asp286Gly (100.0%)		0.25	0.5

Supplementary Table 3. List of lineage 2 samples included in the extended Southern Africa dataset.

Lineage	Sample ID	Accession	Country	Region	Study	Day	Month	Year
2	01_C4006B	SRR9971318	South Africa	Southern Africa	Other AHRI KZN study	13	11	2014
2	01_C4048B	SRR9971120	South Africa	Southern Africa	Other AHRI KZN study	26	3	2014
2	01_C4067B	SRR9971317	South Africa	Southern Africa	Other AHRI KZN study	11	4	2014
2	02_F1069B	SRR11349175	South Africa	Southern Africa	Other AHRI KZN study	14	12	2015
2	02_F1083B	SRR11349164	South Africa	Southern Africa	Other AHRI KZN study	25	1	2016
2	02_F1090B	SRR11349131	South Africa	Southern Africa	Other AHRI KZN study	3	2	2015
2	02_F1110B	SRR9971270	South Africa	Southern Africa	Other AHRI KZN study	4	4	2016
2	02_H0041B	SRR9971081	South Africa	Southern Africa	Other AHRI KZN study	26	10	2016
2	02_H0059B	SRR9971065	South Africa	Southern Africa	Other AHRI KZN study	8	2	2017
2	02_H0067B	SRR9971110	South Africa	Southern Africa	Other AHRI KZN study	30	3	2017
2	02_H0076B	SRR9971307	South Africa	Southern Africa	Other AHRI KZN study	24	5	2017
2	02_H0083B	SRR9971009	South Africa	Southern Africa	Other AHRI KZN study	19	6	2017
2	03_F1021B	SRR11349208	South Africa	Southern Africa	Other AHRI KZN study	24	8	2015
2	03_F1144B	SRR9971271	South Africa	Southern Africa	Other AHRI KZN study	25	7	2016
2	03_H0107B	SRR9971217	South Africa	Southern Africa	Other AHRI KZN study	29	9	2017
2	03_P0040B	SRR9971305	South Africa	Southern Africa	PRAXIS (this study)	4	5	2017
2	03_P0050B	SRR9971087	South Africa	Southern Africa	PRAXIS (this study)	30	5	2017
2	03_P0072B	SRR9971011	South Africa	Southern Africa	PRAXIS (this study)	29	6	2017
2	03_P0082B	SRR9971019	South Africa	Southern Africa	PRAXIS (this study)	12	7	2017
2	03_R0001B	SRR9971076	South Africa	Southern Africa	Other AHRI KZN study	20	12	2016
2	04_H0098B	SRR9971018	South Africa	Southern Africa	Other AHRI KZN study	25	8	2017
2	04_H0106B	SRR9971214	South Africa	Southern Africa	Other AHRI KZN study	21	9	2017
2	04_R0009B	SRR9971064	South Africa	Southern Africa	Other AHRI KZN study	13	2	2017
2	04_R0026B	SRR9971109	South Africa	Southern Africa	Other AHRI KZN study	27	3	2017
2	04_R0030B	SRR9971107	South Africa	Southern Africa	Other AHRI KZN study	7	4	2017
2	04_R0036B	SRR9971108	South Africa	Southern Africa	Other AHRI KZN study	24	4	2017
2	04_R0042B	SRR9971054	South Africa	Southern Africa	Other AHRI KZN study	8	5	2017
2	04_R0043B	SRR9971304	South Africa	Southern Africa	Other AHRI KZN study	8	5	2017
2	04_R0045B	SRR9971037	South Africa	Southern Africa	Other AHRI KZN study	16	5	2017
2	04_R0055B	SRR9971313	South Africa	Southern Africa	Other AHRI KZN study	13	6	2017
2	05_H0027B	SRR9971266	South Africa	Southern Africa	Other AHRI KZN study	4	8	2016
2	05_H0030B	SRR9971268	South Africa	Southern Africa	Other AHRI KZN study	29	8	2016
2	05_H0074B	SRR9971309	South Africa	Southern Africa	Other AHRI KZN study	17	5	2017
2	05_H0111B	SRR9971144	South Africa	Southern Africa	Other AHRI KZN study	2	11	2017
2	05_P00824	SRR9971143	South Africa	Southern Africa	PRAXIS (this study)	7	11	2017
2	05_R0056E	SRR9971016	South Africa	Southern Africa	Other AHRI KZN study	15	6	2017
2	05_R0057B	SRR9971015	South Africa	Southern Africa	Other AHRI KZN study	15	6	2017
2	05_R0059B	SRR9971010	South Africa	Southern Africa	Other AHRI KZN study	21	6	2017
2	05_R0060B	SRR9971012	South Africa	Southern Africa	Other AHRI KZN study	30	6	2017
2	05_R0061B	SRR9971005	South Africa	Southern Africa	Other AHRI KZN study	5	7	2017
2	05_R0064B	SRR9971022	South Africa	Southern Africa	Other AHRI KZN study	14	7	2017
2	05_R0074E	SRR9971025	South Africa	Southern Africa	Other AHRI KZN study	23	8	2017
2	05_R0076E	SRR9971223	South Africa	Southern Africa	Other AHRI KZN study	29	8	2017
2	05_R0087B	SRR9971218	South Africa	Southern Africa	Other AHRI KZN study	9	10	2017
2	05_R0091B	SRR9971208	South Africa	Southern Africa	Other AHRI KZN study	20	10	2017
2	05_R0092B	SRR9971149	South Africa	Southern Africa	Other AHRI KZN study	23	10	2017
2	05_R0094E	SRR11349190	South Africa	Southern Africa	Other AHRI KZN study	31	10	2017
2	05_R0095B	SRR9971145	South Africa	Southern Africa	Other AHRI KZN study	31	10	2017
2	05_R0098B	SRR9971142	South Africa	Southern Africa	Other AHRI KZN study	8	11	2017
2	06_H0032B	SRR9971265	South Africa	Southern Africa	Other AHRI KZN study	29	8	2016
2	06_H0035B	SRR9971264	South Africa	Southern Africa	Other AHRI KZN study	23	9	2016
2	06_H0037B	SRR9971267	South Africa	Southern Africa	Other AHRI KZN study	23	9	2016
2	06_H0042B	SRR9971078	South Africa	Southern Africa	Other AHRI KZN study	26	10	2016
2	06_H0070B	SRR9971116	South Africa	Southern Africa	Other AHRI KZN study	3	5	2017
2	06_H0080B	SRR9971306	South Africa	Southern Africa	Other AHRI KZN study	26	5	2017
2	06_H0086B	SRR9971020	South Africa	Southern Africa	Other AHRI KZN study	6	7	2017
2	06_L00501	SRR9971263	South Africa	Southern Africa	Other AHRI KZN study	6	10	2016
2	06_P0063B	SRR9971085	South Africa	Southern Africa	PRAXIS (this study)	8	6	2017
2	06_P0066B	SRR9971013	South Africa	Southern Africa	PRAXIS (this study)	13	6	2017
2	06_P00825	SRR9971254	South Africa	Southern Africa	PRAXIS (this study)	5	12	2017
2	06_P00826	SRR9971329	South Africa	Southern Africa	PRAXIS (this study)	5	1	2018
2	06_P0085B	SRR9971021	South Africa	Southern Africa	PRAXIS (this study)	19	7	2017
2	06_P01014	SRR9971255	South Africa	Southern Africa	PRAXIS (this study)	5	12	2017
2	06_P0128B	SRR9971220	South Africa	Southern Africa	PRAXIS (this study)	29	9	2017
2	06_P0129B	SRR9971219	South Africa	Southern Africa	PRAXIS (this study)	29	9	2017

2	07_K0005B	SRR9971330	South Africa	Southern Africa	Other AHRI KZN study	19	10	2017
2	07_K0007B	SRR9971148	South Africa	Southern Africa	Other AHRI KZN study	26	10	2017
2	08_H0006B	SRR9971262	South Africa	Southern Africa	Other AHRI KZN study	17	2	2016
2	08_H0021B	SRR9971269	South Africa	Southern Africa	Other AHRI KZN study	9	5	2016
2	08_H0046B	SRR9971079	South Africa	Southern Africa	Other AHRI KZN study	16	11	2016
2	08_H0056B	SRR9971249	South Africa	Southern Africa	Other AHRI KZN study	27	1	2017
2	08_H0088B	SRR9971006	South Africa	Southern Africa	Other AHRI KZN study	6	7	2017
2	08_H0123B	SRR9971324	South Africa	Southern Africa	Other AHRI KZN study	23	1	2018
2	08_H0124B	SRR9971321	South Africa	Southern Africa	Other AHRI KZN study	25	1	2018
2	08_H0125B	SRR9971322	South Africa	Southern Africa	Other AHRI KZN study	25	1	2018
2	08_P0169B	SRR9971257	South Africa	Southern Africa	PRAXIS (this study)	6	12	2017
2	08_P0176B	SRR9971327	South Africa	Southern Africa	PRAXIS (this study)	12	1	2018
2	08_P0179B	SRR9971325	South Africa	Southern Africa	PRAXIS (this study)	19	1	2018
2	08_R0025B	SRR9971112	South Africa	Southern Africa	Other AHRI KZN study	24	3	2017
2	08_R0066B	SRR9971024	South Africa	Southern Africa	Other AHRI KZN study	21	7	2017
2	08_R0067B	SRR9971023	South Africa	Southern Africa	Other AHRI KZN study	25	7	2017
2	09_H0096B	SRR9971222	South Africa	Southern Africa	Other AHRI KZN study	25	8	2017
2	09_H0122B	SRR9971323	South Africa	Southern Africa	Other AHRI KZN study	23	1	2018
2	09_H0129B	SRR9971029	South Africa	Southern Africa	Other AHRI KZN study	5	3	2018
2	09_P0200B	SRR9971028	South Africa	Southern Africa	PRAXIS (this study)	20	2	2018
2	09_R0040B	SRR9971115	South Africa	Southern Africa	Other AHRI KZN study	2	5	2017
2	09_R0069E	SRR9971154	South Africa	Southern Africa	Other AHRI KZN study	11	8	2017
2	09_R0075E	SRR9971017	South Africa	Southern Africa	Other AHRI KZN study	25	8	2017
2	10_H0054B	SRR9971058	South Africa	Southern Africa	Other AHRI KZN study	24	1	2017
2	10_H0065B	SRR9971111	South Africa	Southern Africa	Other AHRI KZN study	24	3	2017
2	10_H0133B	SRR9971034	South Africa	Southern Africa	Other AHRI KZN study	14	3	2018
2	10_H0134B	SRR9971035	South Africa	Southern Africa	Other AHRI KZN study	22	3	2018
2	10_H0136B	SRR9971088	South Africa	Southern Africa	Other AHRI KZN study	16	4	2018
2	10_H0138B	SRR9971089	South Africa	Southern Africa	Other AHRI KZN study	16	4	2018
2	10_K0025B	SRR9971030	South Africa	Southern Africa	Other AHRI KZN study	9	2	2018
2	10_K0026B	SRR9971031	South Africa	Southern Africa	Other AHRI KZN study	14	2	2018
2	10_R0099B	SRR9971156	South Africa	Southern Africa	Other AHRI KZN study	10	11	2017
2	10_R0100B	SRR9971155	South Africa	Southern Africa	Other AHRI KZN study	20	11	2017
2	11_H0001B	SRR9971260	South Africa	Southern Africa	Other AHRI KZN study	21	1	2016
2	11_H0119B	SRR9971259	South Africa	Southern Africa	Other AHRI KZN study	7	12	2017
2	11_H0141B	SRR9971353	South Africa	Southern Africa	Other AHRI KZN study	14	5	2018
2	11_K0009B	SRR9971256	South Africa	Southern Africa	Other AHRI KZN study	5	12	2017
2	11_K0010B	SRR11349182	South Africa	Southern Africa	Other AHRI KZN study	7	12	2017
2	11_K0013B	SRR9971328	South Africa	Southern Africa	Other AHRI KZN study	10	1	2018
2	11_K0021B	SRR11349180	South Africa	Southern Africa	Other AHRI KZN study	31	1	2018
2	11_P0155B	SRR9971147	South Africa	Southern Africa	PRAXIS (this study)	31	10	2017
2	11_P0165B	SRR9971253	South Africa	Southern Africa	PRAXIS (this study)	27	11	2017
2	12_F8103B	SRR9971354	South Africa	Southern Africa	Other AHRI KZN study	10	5	2018
2	12_K0057B	SRR9971356	South Africa	Southern Africa	Other AHRI KZN study	6	6	2018
2	12_P3004B	SRR9971355	South Africa	Southern Africa	PRAXIS (this study)	7	6	2018
2	12_P3008B	SRR9971357	South Africa	Southern Africa	PRAXIS (this study)	19	6	2018
2	12_P3010B	SRR9971350	South Africa	Southern Africa	PRAXIS (this study)	27	6	2018
2	13_F8064B	SRR9971308	South Africa	Southern Africa	Other AHRI KZN study	23	5	2017
2	13_F8078B	SRR9971213	South Africa	Southern Africa	Other AHRI KZN study	31	8	2017
2	13_K0036B	SRR9971032	South Africa	Southern Africa	Other AHRI KZN study	27	3	2018
2	13_K0040B	SRR9971033	South Africa	Southern Africa	Other AHRI KZN study	10	4	2018
2	13_K0045B	SRR9971352	South Africa	Southern Africa	Other AHRI KZN study	8	5	2018
2	13_K0060B	SRR9971349	South Africa	Southern Africa	Other AHRI KZN study	27	6	2018
2	13_K0062B	SRR9971140	South Africa	Southern Africa	Other AHRI KZN study	9	7	2018
2	14_F8102B	SRR9971351	South Africa	Southern Africa	Other AHRI KZN study	8	5	2018
2	14_H0161B	SRR11349173	South Africa	Southern Africa	Other AHRI KZN study	8	8	2018
2	14_H0166B	SRR9971211	South Africa	Southern Africa	Other AHRI KZN study	29	8	2018
2	14_K0016B	SRR9971326	South Africa	Southern Africa	Other AHRI KZN study	16	1	2018
2	14_P02006	SRR11349170	South Africa	Southern Africa	PRAXIS (this study)	3	8	2018
2	14_P3019B	SRR9971205	South Africa	Southern Africa	PRAXIS (this study)	24	8	2018
2	14_P3410B	SRR9971135	South Africa	Southern Africa	PRAXIS (this study)	18	7	2018
2	14_P3510B	SRR9971138	South Africa	Southern Africa	PRAXIS (this study)	28	6	2018
2	15_H0157B	SRR11349169	South Africa	Southern Africa	Other AHRI KZN study	24	7	2018
2	15_H0163B	SRR11349168	South Africa	Southern Africa	Other AHRI KZN study	16	8	2018
2	15_K0061B	SRR9971139	South Africa	Southern Africa	Other AHRI KZN study	3	7	2018
2	15_K0063B	SRR9971134	South Africa	Southern Africa	Other AHRI KZN study	17	7	2018

2	15_K0064B	SRR9971136	South Africa	Southern Africa	Other AHRI KZN study	18	7	2018
2	15_K0065B	SRR9971137	South Africa	Southern Africa	Other AHRI KZN study	19	7	2018
2	15_K0071B	SRR11349159	South Africa	Southern Africa	Other AHRI KZN study	24	8	2018
2	16_R0017B	SRR9971113	South Africa	Southern Africa	Other AHRI KZN study	10	3	2017
2	16_R0020B	SRR9971114	South Africa	Southern Africa	Other AHRI KZN study	15	3	2017
2	21_P3029B	SRR9971060	South Africa	Southern Africa	PRAXIS (this study)	5	10	2018
2	21_P3418B	SRR9971216	South Africa	Southern Africa	PRAXIS (this study)	25	9	2018
2	21_P35242	SRR9971062	South Africa	Southern Africa	PRAXIS (this study)	6	11	2018
2	21_P3524B	SRR9971210	South Africa	Southern Africa	PRAXIS (this study)	5	9	2018
2	22_H0039B	SRR9971080	South Africa	Southern Africa	Other AHRI KZN study	12	10	2016
2	22_K00562	SRR9971358	South Africa	Southern Africa	Other AHRI KZN study	18	6	2018
2	22_K00566	SRR9971141	South Africa	Southern Africa	Other AHRI KZN study	16	7	2018
2	22_K0077B	SRR9971209	South Africa	Southern Africa	Other AHRI KZN study	20	9	2018
2	22_P3415B	SRR9971206	South Africa	Southern Africa	PRAXIS (this study)	17	8	2018
2	23_H0183B	SRR11349151	South Africa	Southern Africa	Other AHRI KZN study	29	11	2018
2	23_H0184B	SRR9971248	South Africa	Southern Africa	Other AHRI KZN study	29	11	2018
2	23_H0190B	SRR9971247	South Africa	Southern Africa	Other AHRI KZN study	12	12	2018
2	23_K0079B	SRR9971215	South Africa	Southern Africa	Other AHRI KZN study	2	10	2018
2	23_K0085B	SRR9971059	South Africa	Southern Africa	Other AHRI KZN study	15	10	2018
2	ERR1034914	ERR1034914	South Africa	Southern Africa	Publicly available	Missing	Missing	Missing
2	ERR1633777	ERR1633777	South Africa	Southern Africa	Publicly available	1	7	2010
2	ERR1633791	ERR1633791	South Africa	Southern Africa	Publicly available	1	7	2010
2	ERR1633796	ERR1633796	South Africa	Southern Africa	Publicly available	1	7	2010
2	ERR1633800	ERR1633800	South Africa	Southern Africa	Publicly available	1	7	2010
2	ERR1633809	ERR1633809	South Africa	Southern Africa	Publicly available	1	7	2010
2	ERR1633816	ERR1633816	South Africa	Southern Africa	Publicly available	1	7	2010
2	ERR1633819	ERR1633819	South Africa	Southern Africa	Publicly available	1	7	2010
2	ERR1633822	ERR1633822	South Africa	Southern Africa	Publicly available	1	7	2010
2	ERR1633823	ERR1633823	South Africa	Southern Africa	Publicly available	1	7	2010
2	ERR1633824	ERR1633824	South Africa	Southern Africa	Publicly available	1	7	2010
2	ERR1633828	ERR1633828	South Africa	Southern Africa	Publicly available	1	7	2010
2	ERR1633829	ERR1633829	South Africa	Southern Africa	Publicly available	1	7	2010
2	ERR1633831	ERR1633831	South Africa	Southern Africa	Publicly available	1	7	2010
2	ERR1633835	ERR1633835	South Africa	Southern Africa	Publicly available	1	7	2010
2	ERR1633844	ERR1633844	South Africa	Southern Africa	Publicly available	1	7	2010
2	ERR1633868	ERR1633868	South Africa	Southern Africa	Publicly available	1	7	2010
2	ERR1633872	ERR1633872	South Africa	Southern Africa	Publicly available	1	7	2010
2	ERR1633881	ERR1633881	South Africa	Southern Africa	Publicly available	1	7	2010
2	ERR1633882	ERR1633882	South Africa	Southern Africa	Publicly available	1	7	2010
2	ERR1633885	ERR1633885	South Africa	Southern Africa	Publicly available	1	7	2010
2	ERR1633895	ERR1633895	South Africa	Southern Africa	Publicly available	1	7	2010
2	ERR1633896	ERR1633896	South Africa	Southern Africa	Publicly available	1	7	2010
2	ERR1633915	ERR1633915	South Africa	Southern Africa	Publicly available	1	7	2010
2	ERR1633922	ERR1633922	South Africa	Southern Africa	Publicly available	1	7	2010
2	ERR1633925	ERR1633925	South Africa	Southern Africa	Publicly available	1	7	2010
2	ERR1633928	ERR1633928	South Africa	Southern Africa	Publicly available	1	7	2010
2	ERR1633933	ERR1633933	South Africa	Southern Africa	Publicly available	1	7	2010
2	ERR1633938	ERR1633938	South Africa	Southern Africa	Publicly available	1	7	2010
2	ERR1633939	ERR1633939	South Africa	Southern Africa	Publicly available	1	7	2010
2	ERR1633944	ERR1633944	South Africa	Southern Africa	Publicly available	1	7	2010
2	ERR1633946	ERR1633946	South Africa	Southern Africa	Publicly available	1	7	2010
2	ERR1633948	ERR1633948	South Africa	Southern Africa	Publicly available	1	7	2010
2	ERR1633951	ERR1633951	South Africa	Southern Africa	Publicly available	1	7	2010
2	ERR1633955	ERR1633955	South Africa	Southern Africa	Publicly available	1	7	2010
2	ERR1633956	ERR1633956	South Africa	Southern Africa	Publicly available	1	7	2010
2	ERR1633962	ERR1633962	South Africa	Southern Africa	Publicly available	1	7	2010
2	ERR1633964	ERR1633964	South Africa	Southern Africa	Publicly available	1	7	2010
2	ERR1873389	ERR1873389	South Africa	Southern Africa	Publicly available	1	7	2009
2	ERR1873390	ERR1873390	South Africa	Southern Africa	Publicly available	1	7	2009
2	ERR1873394	ERR1873394	South Africa	Southern Africa	Publicly available	1	7	2009
2	ERR1873395	ERR1873395	South Africa	Southern Africa	Publicly available	1	7	2008
2	ERR1873397	ERR1873397	South Africa	Southern Africa	Publicly available	1	7	2010
2	ERR1873398	ERR1873398	South Africa	Southern Africa	Publicly available	1	7	2010
2	ERR1873399	ERR1873399	South Africa	Southern Africa	Publicly available	1	7	2010
2	ERR1873402	ERR1873402	South Africa	Southern Africa	Publicly available	1	7	2011
2	ERR1873403	ERR1873403	South Africa	Southern Africa	Publicly available	1	7	2011

2	ERR1873404	ERR1873404	South Africa	Southern Africa	Publicly available	1	7	2011
2	ERR1873407	ERR1873407	South Africa	Southern Africa	Publicly available	1	7	2011
2	ERR1873409	ERR1873409	South Africa	Southern Africa	Publicly available	1	7	2009
2	ERR1873410	ERR1873410	South Africa	Southern Africa	Publicly available	1	7	2010
2	ERR1873412	ERR1873412	South Africa	Southern Africa	Publicly available	1	7	2010
2	ERR1873413	ERR1873413	South Africa	Southern Africa	Publicly available	1	7	2010
2	ERR1873414	ERR1873414	South Africa	Southern Africa	Publicly available	1	7	2010
2	ERR1873415	ERR1873415	South Africa	Southern Africa	Publicly available	1	7	2011
2	ERR1873416	ERR1873416	South Africa	Southern Africa	Publicly available	1	7	2010
2	ERR1873417	ERR1873417	South Africa	Southern Africa	Publicly available	1	7	2011
2	ERR1873421	ERR1873421	South Africa	Southern Africa	Publicly available	1	7	2009
2	ERR1873422	ERR1873422	South Africa	Southern Africa	Publicly available	1	7	2010
2	ERR1873424	ERR1873424	South Africa	Southern Africa	Publicly available	1	7	2010
2	ERR1873426	ERR1873426	South Africa	Southern Africa	Publicly available	1	7	2009
2	ERR1873428	ERR1873428	South Africa	Southern Africa	Publicly available	1	7	2010
2	ERR1873429	ERR1873429	South Africa	Southern Africa	Publicly available	1	7	2010
2	ERR1873430	ERR1873430	South Africa	Southern Africa	Publicly available	1	7	2010
2	ERR1873431	ERR1873431	South Africa	Southern Africa	Publicly available	1	7	2009
2	ERR1873432	ERR1873432	South Africa	Southern Africa	Publicly available	1	7	2010
2	ERR1873434	ERR1873434	South Africa	Southern Africa	Publicly available	1	7	2010
2	ERR1873436	ERR1873436	South Africa	Southern Africa	Publicly available	1	7	2010
2	ERR1873438	ERR1873438	South Africa	Southern Africa	Publicly available	1	7	2011
2	ERR1873439	ERR1873439	South Africa	Southern Africa	Publicly available	1	7	2011
2	ERR1873440	ERR1873440	South Africa	Southern Africa	Publicly available	1	7	2011
2	ERR1873442	ERR1873442	South Africa	Southern Africa	Publicly available	1	7	2010
2	ERR1873443	ERR1873443	South Africa	Southern Africa	Publicly available	1	7	2010
2	ERR1873445	ERR1873445	South Africa	Southern Africa	Publicly available	1	7	2010
2	ERR1873447	ERR1873447	South Africa	Southern Africa	Publicly available	1	7	2010
2	ERR1873450	ERR1873450	South Africa	Southern Africa	Publicly available	1	7	2010
2	ERR1873451	ERR1873451	South Africa	Southern Africa	Publicly available	1	7	2010
2	ERR1873452	ERR1873452	South Africa	Southern Africa	Publicly available	1	7	2010
2	ERR1873453	ERR1873453	South Africa	Southern Africa	Publicly available	1	7	2010
2	ERR1873454	ERR1873454	South Africa	Southern Africa	Publicly available	1	7	2010
2	ERR1873455	ERR1873455	South Africa	Southern Africa	Publicly available	1	7	2010
2	ERR1873456	ERR1873456	South Africa	Southern Africa	Publicly available	1	7	2010
2	ERR1873457	ERR1873457	South Africa	Southern Africa	Publicly available	1	7	2010
2	ERR1873458	ERR1873458	South Africa	Southern Africa	Publicly available	1	7	2010
2	ERR1873460	ERR1873460	South Africa	Southern Africa	Publicly available	1	7	2010
2	ERR1873461	ERR1873461	South Africa	Southern Africa	Publicly available	1	7	2010
2	ERR1873463	ERR1873463	South Africa	Southern Africa	Publicly available	1	7	2011
2	ERR1873464	ERR1873464	South Africa	Southern Africa	Publicly available	1	7	2011
2	ERR1873465	ERR1873465	South Africa	Southern Africa	Publicly available	1	7	2011
2	ERR1873466	ERR1873466	South Africa	Southern Africa	Publicly available	1	7	2011
2	ERR1873467	ERR1873467	South Africa	Southern Africa	Publicly available	1	7	2011
2	ERR1873468	ERR1873468	South Africa	Southern Africa	Publicly available	1	7	2011
2	ERR1873469	ERR1873469	South Africa	Southern Africa	Publicly available	1	7	2011
2	ERR1873470	ERR1873470	South Africa	Southern Africa	Publicly available	1	7	2011
2	ERR1873471	ERR1873471	South Africa	Southern Africa	Publicly available	1	7	2011
2	ERR1873472	ERR1873472	South Africa	Southern Africa	Publicly available	1	7	2011
2	ERR1873473	ERR1873473	South Africa	Southern Africa	Publicly available	1	7	2011
2	ERR1873474	ERR1873474	South Africa	Southern Africa	Publicly available	1	7	2011
2	ERR1873476	ERR1873476	South Africa	Southern Africa	Publicly available	1	7	2011
2	ERR1873477	ERR1873477	South Africa	Southern Africa	Publicly available	1	7	2011
2	ERR1873478	ERR1873478	South Africa	Southern Africa	Publicly available	1	7	2011
2	ERR1873479	ERR1873479	South Africa	Southern Africa	Publicly available	1	7	2011
2	ERR1873481	ERR1873481	South Africa	Southern Africa	Publicly available	1	7	2011
2	ERR1873482	ERR1873482	South Africa	Southern Africa	Publicly available	1	7	2012
2	ERR1873484	ERR1873484	South Africa	Southern Africa	Publicly available	1	7	2011
2	ERR1873485	ERR1873485	South Africa	Southern Africa	Publicly available	1	7	2012
2	ERR1873486	ERR1873486	South Africa	Southern Africa	Publicly available	1	7	2011
2	ERR1873487	ERR1873487	South Africa	Southern Africa	Publicly available	1	7	2011
2	ERR1873488	ERR1873488	South Africa	Southern Africa	Publicly available	1	7	2012
2	ERR1873489	ERR1873489	South Africa	Southern Africa	Publicly available	1	7	2011
2	ERR1873490	ERR1873490	South Africa	Southern Africa	Publicly available	1	7	2012
2	ERR1873491	ERR1873491	South Africa	Southern Africa	Publicly available	1	7	2012
2	ERR1873493	ERR1873493	South Africa	Southern Africa	Publicly available	1	7	2012

2	ERR1873494	ERR1873494	South Africa	Southern Africa	Publicly available	1	7	2012
2	ERR1873495	ERR1873495	South Africa	Southern Africa	Publicly available	1	7	2012
2	ERR1873496	ERR1873496	South Africa	Southern Africa	Publicly available	1	7	2012
2	ERR1873497	ERR1873497	South Africa	Southern Africa	Publicly available	1	7	2012
2	ERR1873498	ERR1873498	South Africa	Southern Africa	Publicly available	1	7	2012
2	ERR1873499	ERR1873499	South Africa	Southern Africa	Publicly available	1	7	2012
2	ERR1873500	ERR1873500	South Africa	Southern Africa	Publicly available	1	7	2012
2	ERR1873501	ERR1873501	South Africa	Southern Africa	Publicly available	1	7	2012
2	ERR1873502	ERR1873502	South Africa	Southern Africa	Publicly available	1	7	2012
2	ERR1873503	ERR1873503	South Africa	Southern Africa	Publicly available	1	7	2012
2	ERR1873504	ERR1873504	South Africa	Southern Africa	Publicly available	1	7	2012
2	ERR1873505	ERR1873505	South Africa	Southern Africa	Publicly available	1	7	2012
2	ERR1873507	ERR1873507	South Africa	Southern Africa	Publicly available	1	7	2012
2	ERR1873508	ERR1873508	South Africa	Southern Africa	Publicly available	1	7	2012
2	ERR1873509	ERR1873509	South Africa	Southern Africa	Publicly available	1	7	2012
2	ERR1873510	ERR1873510	South Africa	Southern Africa	Publicly available	1	7	2012
2	ERR1873514	ERR1873514	South Africa	Southern Africa	Publicly available	1	7	2012
2	ERR1873515	ERR1873515	South Africa	Southern Africa	Publicly available	1	7	2012
2	ERR1873516	ERR1873516	South Africa	Southern Africa	Publicly available	1	7	2012
2	ERR1873519	ERR1873519	South Africa	Southern Africa	Publicly available	1	7	2012
2	ERR1873520	ERR1873520	South Africa	Southern Africa	Publicly available	1	7	2012
2	ERR1873521	ERR1873521	South Africa	Southern Africa	Publicly available	1	7	2008
2	ERR1873522	ERR1873522	South Africa	Southern Africa	Publicly available	1	7	2008
2	ERR1873523	ERR1873523	South Africa	Southern Africa	Publicly available	1	7	2008
2	ERR1873524	ERR1873524	South Africa	Southern Africa	Publicly available	1	7	2008
2	ERR1873525	ERR1873525	South Africa	Southern Africa	Publicly available	1	7	2008
2	ERR1873526	ERR1873526	South Africa	Southern Africa	Publicly available	1	7	2008
2	ERR1873527	ERR1873527	South Africa	Southern Africa	Publicly available	1	7	2008
2	ERR1873528	ERR1873528	South Africa	Southern Africa	Publicly available	1	7	2008
2	ERR1873529	ERR1873529	South Africa	Southern Africa	Publicly available	1	7	2008
2	ERR1873530	ERR1873530	South Africa	Southern Africa	Publicly available	1	7	2009
2	ERR1873532	ERR1873532	South Africa	Southern Africa	Publicly available	1	7	2009
2	ERR1873533	ERR1873533	South Africa	Southern Africa	Publicly available	1	7	2009
2	ERR1873535	ERR1873535	South Africa	Southern Africa	Publicly available	1	7	2009
2	ERR1873536	ERR1873536	South Africa	Southern Africa	Publicly available	1	7	2009
2	ERR1873537	ERR1873537	South Africa	Southern Africa	Publicly available	1	7	2009
2	ERR1873538	ERR1873538	South Africa	Southern Africa	Publicly available	1	7	2009
2	ERR1873539	ERR1873539	South Africa	Southern Africa	Publicly available	1	7	2009
2	ERR1873541	ERR1873541	South Africa	Southern Africa	Publicly available	1	7	2009
2	ERR1873542	ERR1873542	South Africa	Southern Africa	Publicly available	1	7	2009
2	ERR1873543	ERR1873543	South Africa	Southern Africa	Publicly available	1	7	2009
2	ERR1873544	ERR1873544	South Africa	Southern Africa	Publicly available	1	7	2009
2	ERR1873545	ERR1873545	South Africa	Southern Africa	Publicly available	1	7	2009
2	ERR1873547	ERR1873547	South Africa	Southern Africa	Publicly available	1	7	2009
2	ERR1873548	ERR1873548	South Africa	Southern Africa	Publicly available	1	7	2009
2	ERR1873551	ERR1873551	South Africa	Southern Africa	Publicly available	1	7	2009
2	ERR1873552	ERR1873552	South Africa	Southern Africa	Publicly available	1	7	2009
2	ERR1873553	ERR1873553	South Africa	Southern Africa	Publicly available	1	7	2010
2	ERR1873555	ERR1873555	South Africa	Southern Africa	Publicly available	1	7	2010
2	ERR1873556	ERR1873556	South Africa	Southern Africa	Publicly available	1	7	2010
2	ERR1873557	ERR1873557	South Africa	Southern Africa	Publicly available	1	7	2010
2	ERR1873558	ERR1873558	South Africa	Southern Africa	Publicly available	1	7	2010
2	ERR1873559	ERR1873559	South Africa	Southern Africa	Publicly available	1	7	2010
2	ERR1873560	ERR1873560	South Africa	Southern Africa	Publicly available	1	7	2010
2	ERR1873561	ERR1873561	South Africa	Southern Africa	Publicly available	1	7	2010
2	ERR1873562	ERR1873562	South Africa	Southern Africa	Publicly available	1	7	2010
2	ERR1873563	ERR1873563	South Africa	Southern Africa	Publicly available	1	7	2010
2	ERR1873564	ERR1873564	South Africa	Southern Africa	Publicly available	1	7	2010
2	ERR1873565	ERR1873565	South Africa	Southern Africa	Publicly available	1	7	2010
2	ERR190363	ERR190363	Malawi	Southern Africa	Publicly available	1	7	2002
2	ERR190368	ERR190368	Malawi	Southern Africa	Publicly available	1	7	2001
2	ERR190369	ERR190369	Malawi	Southern Africa	Publicly available	1	7	2002
2	ERR190378	ERR190378	Malawi	Southern Africa	Publicly available	1	7	1999
2	ERR190386	ERR190386	Malawi	Southern Africa	Publicly available	1	7	1998
2	ERR190401	ERR190401	Malawi	Southern Africa	Publicly available	1	7	2002
2	ERR1988847	ERR1988847	South Africa	Southern Africa	Publicly available	1	7	2010

2	ERR550739	ERR550739	South Africa	Southern Africa	Publicly available	1	7	2004
2	ERR551071	ERR551071	eSwatini	Southern Africa	Publicly available	1	7	2009
2	ERR551079	ERR551079	eSwatini	Southern Africa	Publicly available	1	7	2009
2	ERR551854	ERR551854	eSwatini	Southern Africa	Publicly available	1	7	2009
2	ERR552743	ERR552743	eSwatini	Southern Africa	Publicly available	1	7	2009
2	ERR553116	ERR553116	eSwatini	Southern Africa	Publicly available	1	7	2009
2	ERR553156	ERR553156	eSwatini	Southern Africa	Publicly available	1	7	2009
2	ERR553313	ERR553313	eSwatini	Southern Africa	Publicly available	1	7	2009
2	ERR553324	ERR553324	eSwatini	Southern Africa	Publicly available	1	7	2009
2	KR_F1051B	SRR9971075	South Africa	Southern Africa	Other AHRI KZN study	9	11	2015
2	KR_F1056B	SRR11349132	South Africa	Southern Africa	Other AHRI KZN study	16	11	2015
2	KR_F1061B	SRR11349207	South Africa	Southern Africa	Other AHRI KZN study	30	11	2015
2	KR_F1066B	SRR11349205	South Africa	Southern Africa	Other AHRI KZN study	9	12	2015
2	KR_F1067B	SRR11349204	South Africa	Southern Africa	Other AHRI KZN study	9	12	2015
2	KR_F1126B	SRR11349196	South Africa	Southern Africa	Other AHRI KZN study	30	5	2016
2	SRR1019146	SRR1019146	South Africa	Southern Africa	Publicly available	1	7	2013
2	SRR1140900	SRR1140900	South Africa	Southern Africa	Publicly available	1	7	2013
2	SRR1140901	SRR1140901	South Africa	Southern Africa	Publicly available	1	7	2013
2	SRR1140916	SRR1140916	South Africa	Southern Africa	Publicly available	1	7	2013
2	SRR1140928	SRR1140928	South Africa	Southern Africa	Publicly available	1	7	2013
2	SRR1140940	SRR1140940	South Africa	Southern Africa	Publicly available	1	7	2013
2	SRR1140947	SRR1140947	South Africa	Southern Africa	Publicly available	1	7	2013
2	SRR1140948	SRR1140948	South Africa	Southern Africa	Publicly available	1	7	2013
2	SRR1140964	SRR1140964	South Africa	Southern Africa	Publicly available	1	7	2013
2	SRR1167167	SRR1167167	South Africa	Southern Africa	Publicly available	Missing	Missing	Missing
2	SRR1173130	SRR1173130	South Africa	Southern Africa	Publicly available	1	7	2013
2	SRR1173289	SRR1173289	South Africa	Southern Africa	Publicly available	Missing	Missing	Missing
2	SRR1173814	SRR1173814	South Africa	Southern Africa	Publicly available	Missing	Missing	Missing
2	SRR1173850	SRR1173850	South Africa	Southern Africa	Publicly available	Missing	Missing	Missing
2	SRR1175054	SRR1175054	South Africa	Southern Africa	Publicly available	1	7	2013
2	SRR1175358	SRR1175358	South Africa	Southern Africa	Publicly available	1	7	2013
2	SRR1180352	SRR1180352	South Africa	Southern Africa	Publicly available	1	7	2013
2	SRR1180357	SRR1180357	South Africa	Southern Africa	Publicly available	1	7	2013
2	SRR1180378	SRR1180378	South Africa	Southern Africa	Publicly available	1	7	2013
2	SRR1180385	SRR1180385	South Africa	Southern Africa	Publicly available	1	7	2013
2	SRR1180396	SRR1180396	South Africa	Southern Africa	Publicly available	1	7	2013
2	SRR1180398	SRR1180398	South Africa	Southern Africa	Publicly available	1	7	2013
2	SRR1180400	SRR1180400	South Africa	Southern Africa	Publicly available	1	7	2013
2	SRR1180404	SRR1180404	South Africa	Southern Africa	Publicly available	1	7	2013
2	SRR1180421	SRR1180421	South Africa	Southern Africa	Publicly available	1	7	2013
2	SRR1180427	SRR1180427	South Africa	Southern Africa	Publicly available	1	7	2013
2	SRR1180432	SRR1180432	South Africa	Southern Africa	Publicly available	1	7	2013
2	SRR1180434	SRR1180434	South Africa	Southern Africa	Publicly available	1	7	2013
2	SRR1180442	SRR1180442	South Africa	Southern Africa	Publicly available	1	7	2013
2	SRR1182970	SRR1182970	South Africa	Southern Africa	Publicly available	1	7	2013
2	SRR1182987	SRR1182987	South Africa	Southern Africa	Publicly available	1	7	2013
2	SRR1183012	SRR1183012	South Africa	Southern Africa	Publicly available	1	7	2013
2	SRR1183048	SRR1183048	South Africa	Southern Africa	Publicly available	1	7	2013
2	SRR1183082	SRR1183082	South Africa	Southern Africa	Publicly available	1	7	2011
2	SRR1183089	SRR1183089	South Africa	Southern Africa	Publicly available	1	7	2013
2	SRR1183095	SRR1183095	South Africa	Southern Africa	Publicly available	1	7	2013
2	SRR1183120	SRR1183120	South Africa	Southern Africa	Publicly available	1	7	2013
2	SRR1183136	SRR1183136	South Africa	Southern Africa	Publicly available	1	7	2013
2	SRR1184296	SRR1184296	South Africa	Southern Africa	Publicly available	1	7	2013
2	SRR1184303	SRR1184303	South Africa	Southern Africa	Publicly available	1	7	2013
2	SRR1184307	SRR1184307	South Africa	Southern Africa	Publicly available	1	7	2013
2	SRR1184311	SRR1184311	South Africa	Southern Africa	Publicly available	1	7	2013
2	SRR1184318	SRR1184318	South Africa	Southern Africa	Publicly available	1	7	2013
2	SRR1184323	SRR1184323	South Africa	Southern Africa	Publicly available	1	7	2013
2	SRR1184330	SRR1184330	South Africa	Southern Africa	Publicly available	1	7	2013
2	SRR1184332	SRR1184332	South Africa	Southern Africa	Publicly available	1	7	2013
2	SRR1184338	SRR1184338	South Africa	Southern Africa	Publicly available	1	7	2013
2	SRR1184344	SRR1184344	South Africa	Southern Africa	Publicly available	1	7	2013
2	SRR1184364	SRR1184364	South Africa	Southern Africa	Publicly available	1	7	2013
2	SRR1184365	SRR1184365	South Africa	Southern Africa	Publicly available	1	7	2013
2	SRR1184370	SRR1184370	South Africa	Southern Africa	Publicly available	1	7	2013

2	SRR1184373	SRR1184373	South Africa	Southern Africa	Publicly available	1	7	2013
2	SRR1184376	SRR1184376	South Africa	Southern Africa	Publicly available	1	7	2013
2	SRR1184386	SRR1184386	South Africa	Southern Africa	Publicly available	1	7	2013
2	SRR1184387	SRR1184387	South Africa	Southern Africa	Publicly available	1	7	2013
2	SRR2101676	SRR2101676	South Africa	Southern Africa	Publicly available	18	5	2013
2	SRR6207273	SRR6207273	South Africa	Southern Africa	Publicly available	1	7	2012
2	SRR6207277	SRR6207277	South Africa	Southern Africa	Publicly available	1	7	2012
2	SRR6207279	SRR6207279	South Africa	Southern Africa	Publicly available	1	7	2012
2	SRR6207281	SRR6207281	South Africa	Southern Africa	Publicly available	1	7	2012
2	SRR6207285	SRR6207285	South Africa	Southern Africa	Publicly available	1	7	2012
2	SRR6207286	SRR6207286	South Africa	Southern Africa	Publicly available	1	7	2012
2	SRR832982	SRR832982	South Africa	Southern Africa	Publicly available	1	7	2008
2	SRR832985	SRR832985	South Africa	Southern Africa	Publicly available	1	7	2008
2	SRR832998	SRR832998	South Africa	Southern Africa	Publicly available	1	7	2008
2	SRR833001	SRR833001	South Africa	Southern Africa	Publicly available	1	7	2008
2	SRR833007	SRR833007	South Africa	Southern Africa	Publicly available	1	7	2008
2	SRR833010	SRR833010	South Africa	Southern Africa	Publicly available	1	7	2010
2	SRR833043	SRR833043	South Africa	Southern Africa	Publicly available	1	7	2010
2	SRR833057	SRR833057	South Africa	Southern Africa	Publicly available	1	7	2010
2	SRR833061	SRR833061	South Africa	Southern Africa	Publicly available	1	7	2008
2	SRR833092	SRR833092	South Africa	Southern Africa	Publicly available	1	7	2010
2	SRR833112	SRR833112	South Africa	Southern Africa	Publicly available	1	7	2008
2	SRR833133	SRR833133	South Africa	Southern Africa	Publicly available	1	7	2009
2	SRR833142	SRR833142	South Africa	Southern Africa	Publicly available	1	7	2010
2	SRR833143	SRR833143	South Africa	Southern Africa	Publicly available	1	7	2008
2	SRR833145	SRR833145	South Africa	Southern Africa	Publicly available	1	7	2010
2	SRR833147	SRR833147	South Africa	Southern Africa	Publicly available	1	7	2008
2	SRR833190	SRR833190	South Africa	Southern Africa	Publicly available	1	7	2010
2	SRR833192	SRR833192	South Africa	Southern Africa	Publicly available	1	7	2010
2	SRR847786	SRR847786	South Africa	Southern Africa	Publicly available	1	7	2012
2	SRR847799	SRR847799	South Africa	Southern Africa	Publicly available	1	7	2011
2	SRR958198	SRR958198	South Africa	Southern Africa	Publicly available	1	7	2008
2	SRR958231	SRR958231	South Africa	Southern Africa	Publicly available	1	7	2008
2	SRR958234	SRR958234	South Africa	Southern Africa	Publicly available	1	7	2008
2	SRR958237	SRR958237	South Africa	Southern Africa	Publicly available	1	7	2008

Supplementary Table 4. List of lineage 4 samples included in the extended Southern Africa dataset.

Lineage	Sample ID	Accession	Country	Study	Region	Day	Month	Year
4	01_C0067B	SRR9971303	South Africa	Other AHRI KZN study	Southern Africa	22	10	2013
4	01_C4027B	SRR9971302	South Africa	Other AHRI KZN study	Southern Africa	26	2	2014
4	01_F1045B	SRR11349211	South Africa	Other AHRI KZN study	Southern Africa	10	7	2015
4	01_F1049B	SRR11349210	South Africa	Other AHRI KZN study	Southern Africa	19	10	2015
4	01_F1064B	SRR9971298	South Africa	Other AHRI KZN study	Southern Africa	12	2	2015
4	01_F1098B	SRR11349186	South Africa	Other AHRI KZN study	Southern Africa	29	2	2016
4	01_P00271	SRR9971044	South Africa	PRAXIS (this study)	Southern Africa	22	3	2017
4	01_P00273	SRR9971043	South Africa	PRAXIS (this study)	Southern Africa	9	6	2017
4	01_P00274	SRR9971042	South Africa	PRAXIS (this study)	Southern Africa	21	6	2017
4	01_P0033B	SRR9971041	South Africa	PRAXIS (this study)	Southern Africa	16	3	2017
4	01_P0034B	SRR9971040	South Africa	PRAXIS (this study)	Southern Africa	29	3	2017
4	01_P0037B	SRR9971039	South Africa	PRAXIS (this study)	Southern Africa	12	4	2017
4	02_F1086B	SRR11349153	South Africa	Other AHRI KZN study	Southern Africa	2	1	2016
4	02_F1091B	SRR9971008	South Africa	Other AHRI KZN study	Southern Africa	2	8	2016
4	02_F1092B	SRR9971310	South Africa	Other AHRI KZN study	Southern Africa	2	10	2016
4	02_F1093B	SRR11349197	South Africa	Other AHRI KZN study	Southern Africa	2	10	2016
4	02_F1097B	SRR9971204	South Africa	Other AHRI KZN study	Southern Africa	24	2	2016
4	02_H0007B	SRR11349195	South Africa	Other AHRI KZN study	Southern Africa	17	2	2016
4	02_H0045B	SRR9971196	South Africa	Other AHRI KZN study	Southern Africa	11	10	2016
4	02_H0052B	SRR9971195	South Africa	Other AHRI KZN study	Southern Africa	19	12	2016
4	02_H0066B	SRR9971038	South Africa	Other AHRI KZN study	Southern Africa	29	3	2017
4	02_H0069B	SRR9971360	South Africa	Other AHRI KZN study	Southern Africa	21	4	2017
4	02_H0075B	SRR9971361	South Africa	Other AHRI KZN study	Southern Africa	24	5	2017
4	02_H0077B	SRR9971362	South Africa	Other AHRI KZN study	Southern Africa	24	5	2017
4	03_F1003B	SRR9971301	South Africa	Other AHRI KZN study	Southern Africa	22	6	2014
4	03_F1004B	SRR11349209	South Africa	Other AHRI KZN study	Southern Africa	20	6	2015
4	03_F1082B	SRR11349194	South Africa	Other AHRI KZN study	Southern Africa	25	1	2016
4	03_F1095B	SRR9971197	South Africa	Other AHRI KZN study	Southern Africa	22	2	2016
4	03_F1100B	SRR9971200	South Africa	Other AHRI KZN study	Southern Africa	29	2	2016
4	03_F1124B	SRR9971199	South Africa	Other AHRI KZN study	Southern Africa	25	5	2016
4	03_F1139B	SRR11349193	South Africa	Other AHRI KZN study	Southern Africa	11	7	2016
4	03_F1141B	SRR9971201	South Africa	Other AHRI KZN study	Southern Africa	20	7	2016
4	03_F1143B	SRR11349192	South Africa	Other AHRI KZN study	Southern Africa	20	7	2016
4	03_H0085B	SRR9971363	South Africa	Other AHRI KZN study	Southern Africa	21	6	2017
4	03_H0104B	SRR9971364	South Africa	Other AHRI KZN study	Southern Africa	21	9	2017
4	03_P00056	SRR9971365	South Africa	PRAXIS (this study)	Southern Africa	10	7	2017
4	03_P0005B	SRR9971366	South Africa	PRAXIS (this study)	Southern Africa	23	1	2017
4	03_P0017B	SRR9971367	South Africa	PRAXIS (this study)	Southern Africa	7	2	2017
4	03_P0051B	SRR9971368	South Africa	PRAXIS (this study)	Southern Africa	30	5	2017
4	03_P0061B	SRR9971369	South Africa	PRAXIS (this study)	Southern Africa	8	6	2017
4	03_P0078B	SRR9971186	South Africa	PRAXIS (this study)	Southern Africa	11	7	2017
4	03_P0080B	SRR9971345	South Africa	PRAXIS (this study)	Southern Africa	10	7	2017
4	03_P0081B	SRR9971188	South Africa	PRAXIS (this study)	Southern Africa	12	7	2017
4	03_P0090B	SRR9971187	South Africa	PRAXIS (this study)	Southern Africa	1	8	2017
4	03_P0106B	SRR9971190	South Africa	PRAXIS (this study)	Southern Africa	30	8	2017
4	03_P0117B	SRR9971189	South Africa	PRAXIS (this study)	Southern Africa	21	9	2017
4	04_H0102B	SRR9971192	South Africa	Other AHRI KZN study	Southern Africa	7	9	2017
4	04_H0109B	SRR9971191	South Africa	Other AHRI KZN study	Southern Africa	11	10	2017
4	04_H0110B	SRR9971194	South Africa	Other AHRI KZN study	Southern Africa	24	10	2017
4	04_P00276	SRR9971193	South Africa	PRAXIS (this study)	Southern Africa	18	8	2017
4	04_P0097B	SRR11349191	South Africa	PRAXIS (this study)	Southern Africa	16	8	2017
4	04_P0098B	SRR9971056	South Africa	PRAXIS (this study)	Southern Africa	21	8	2017
4	04_P0101B	SRR9970995	South Africa	PRAXIS (this study)	Southern Africa	22	8	2017
4	04_P0119B	SRR9970996	South Africa	PRAXIS (this study)	Southern Africa	22	9	2017
4	04_P0121B	SRR9971057	South Africa	PRAXIS (this study)	Southern Africa	27	9	2017
4	04_P0133B	SRR9970994	South Africa	PRAXIS (this study)	Southern Africa	5	10	2017
4	04_P0139B	SRR9970999	South Africa	PRAXIS (this study)	Southern Africa	16	10	2017
4	04_P0141B	SRR9971000	South Africa	PRAXIS (this study)	Southern Africa	18	10	2017
4	04_P0152B	SRR9970997	South Africa	PRAXIS (this study)	Southern Africa	31	10	2017
4	04_P0156B	SRR9970998	South Africa	PRAXIS (this study)	Southern Africa	31	10	2017

4	04_R0003B	SRR9971168	South Africa	Other AHRI KZN study	Southern Africa	1	2	2017
4	04_R0005B	SRR9971167	South Africa	Other AHRI KZN study	Southern Africa	3	2	2017
4	04_R0041B	SRR9971162	South Africa	Other AHRI KZN study	Southern Africa	4	5	2017
4	04_R0051B	SRR9971161	South Africa	Other AHRI KZN study	Southern Africa	30	5	2017
4	04_R0052B	SRR9971160	South Africa	Other AHRI KZN study	Southern Africa	5	6	2017
4	05_H0015B	SRR9971125	South Africa	Other AHRI KZN study	Southern Africa	4	5	2016
4	05_H0033B	SRR9971130	South Africa	Other AHRI KZN study	Southern Africa	15	9	2016
4	05_H0079B	SRR9971150	South Africa	Other AHRI KZN study	Southern Africa	26	5	2017
4	05_H0115B	SRR9971319	South Africa	Other AHRI KZN study	Southern Africa	23	11	2017
4	05_P0002B	SRR9971131	South Africa	PRAXIS (this study)	Southern Africa	13	12	2016
4	05_P0044B	SRR9971315	South Africa	PRAXIS (this study)	Southern Africa	19	5	2017
4	05_R0062B	SRR9971071	South Africa	Other AHRI KZN study	Southern Africa	7	7	2017
4	05_R0071B	SRR9971069	South Africa	Other AHRI KZN study	Southern Africa	15	8	2017
4	05_R0073B	SRR9971388	South Africa	Other AHRI KZN study	Southern Africa	21	8	2017
4	05_R0077B	SRR9971389	South Africa	Other AHRI KZN study	Southern Africa	30	8	2017
4	05_R0081B	SRR9971384	South Africa	Other AHRI KZN study	Southern Africa	19	9	2017
4	05_R0083B	SRR9971385	South Africa	Other AHRI KZN study	Southern Africa	21	9	2017
4	05_R0084B	SRR9971386	South Africa	Other AHRI KZN study	Southern Africa	28	9	2017
4	05_R0085B	SRR9971387	South Africa	Other AHRI KZN study	Southern Africa	3	10	2017
4	05_R0086B	SRR9971380	South Africa	Other AHRI KZN study	Southern Africa	4	10	2017
4	05_R0088B	SRR9971381	South Africa	Other AHRI KZN study	Southern Africa	18	10	2017
4	05_R0093B	SRR9971382	South Africa	Other AHRI KZN study	Southern Africa	30	10	2017
4	05_R0097B	SRR9971383	South Africa	Other AHRI KZN study	Southern Africa	3	11	2017
4	06_H0028B	SRR9971132	South Africa	Other AHRI KZN study	Southern Africa	24	8	2016
4	06_H0034B	SRR9971133	South Africa	Other AHRI KZN study	Southern Africa	15	9	2016
4	06_H0061B	SRR9971245	South Africa	Other AHRI KZN study	Southern Africa	3	3	2017
4	06_H0078B	SRR9971244	South Africa	Other AHRI KZN study	Southern Africa	26	5	2017
4	06_L00101	SRR9971126	South Africa	Other AHRI KZN study	Southern Africa	28	1	2016
4	06_L00103	SRR9971241	South Africa	Other AHRI KZN study	Southern Africa	14	8	2017
4	06_L00201	SRR9971127	South Africa	Other AHRI KZN study	Southern Africa	23	11	2016
4	06_L00301	SRR9971128	South Africa	Other AHRI KZN study	Southern Africa	28	11	2016
4	06_L00701	SRR9971240	South Africa	Other AHRI KZN study	Southern Africa	19	4	2017
4	06_L00901	SRR9971243	South Africa	Other AHRI KZN study	Southern Africa	17	5	2017
4	06_P0027B	SRR9971242	South Africa	PRAXIS (this study)	Southern Africa	24	2	2017
4	06_P0060B	SRR9971237	South Africa	PRAXIS (this study)	Southern Africa	8	6	2017
4	06_P0062B	SRR9971236	South Africa	PRAXIS (this study)	Southern Africa	8	6	2017
4	07_H0009B	SRR9971129	South Africa	Other AHRI KZN study	Southern Africa	3	1	2016
4	07_H0011B	SRR9971274	South Africa	Other AHRI KZN study	Southern Africa	3	7	2016
4	07_H0091B	SRR9971239	South Africa	Other AHRI KZN study	Southern Africa	7	12	2017
4	07_H0092B	SRR9971238	South Africa	Other AHRI KZN study	Southern Africa	19	7	2017
4	07_H0093B	SRR9971251	South Africa	Other AHRI KZN study	Southern Africa	19	7	2017
4	07_K0006B	SRR9971252	South Africa	Other AHRI KZN study	Southern Africa	24	10	2017
4	07_K0008B	SRR9971185	South Africa	Other AHRI KZN study	Southern Africa	2	11	2017
4	07_L00102	SRR9971250	South Africa	Other AHRI KZN study	Southern Africa	19	1	2017
4	07_P0004B	SRR9971183	South Africa	PRAXIS (this study)	Southern Africa	19	1	2017
4	07_P0126B	SRR9971184	South Africa	PRAXIS (this study)	Southern Africa	29	9	2017
4	08_H0116B	SRR9971181	South Africa	Other AHRI KZN study	Southern Africa	27	11	2017
4	08_K0028B	SRR9971027	South Africa	Other AHRI KZN study	Southern Africa	22	2	2018
4	08_P0147B	SRR9971182	South Africa	PRAXIS (this study)	Southern Africa	26	10	2017
4	08_P01501	SRR9971179	South Africa	PRAXIS (this study)	Southern Africa	27	11	2017
4	08_P01502	SRR9971180	South Africa	PRAXIS (this study)	Southern Africa	18	12	2017
4	08_P0150B	SRR9971285	South Africa	PRAXIS (this study)	Southern Africa	30	10	2017
4	08_P0161B	SRR9971284	South Africa	PRAXIS (this study)	Southern Africa	20	11	2017
4	08_R0022B	SRR9971293	South Africa	Other AHRI KZN study	Southern Africa	23	3	2017
4	08_R0038B	SRR9971292	South Africa	Other AHRI KZN study	Southern Africa	28	4	2017
4	08_R0046B	SRR9971291	South Africa	Other AHRI KZN study	Southern Africa	17	5	2017
4	08_R0053B	SRR9971290	South Africa	Other AHRI KZN study	Southern Africa	8	6	2017
4	08_R0063B	SRR9971289	South Africa	Other AHRI KZN study	Southern Africa	12	7	2017
4	08_R0065B	SRR9971288	South Africa	Other AHRI KZN study	Southern Africa	20	7	2017
4	09_H0105B	SRR9971286	South Africa	Other AHRI KZN study	Southern Africa	21	9	2017
4	09_H0126B	SRR11349188	South Africa	Other AHRI KZN study	Southern Africa	6	2	2018

4	09_K0017B	SRR9971224	South Africa	Other AHRI KZN study	Southern Africa	18	1	2018
4	09_K0022B	SRR9971341	South Africa	Other AHRI KZN study	Southern Africa	1	2	2018
4	09_P00051	SRR9971105	South Africa	PRAXIS (this study)	Southern Africa	21	2	2017
4	09_P0014B	SRR9971106	South Africa	PRAXIS (this study)	Southern Africa	1	2	2017
4	09_P0084B	SRR9971097	South Africa	PRAXIS (this study)	Southern Africa	19	7	2017
4	09_R0079E	SRR11349185	South Africa	Other AHRI KZN study	Southern Africa	14	9	2017
4	10_H0094B	SRR9971100	South Africa	Other AHRI KZN study	Southern Africa	27	7	2017
4	10_H0132B	SRR9971342	South Africa	Other AHRI KZN study	Southern Africa	20	3	2018
4	10_H0140B	SRR9971343	South Africa	Other AHRI KZN study	Southern Africa	3	5	2018
4	10_P0023B	SRR9971101	South Africa	PRAXIS (this study)	Southern Africa	17	2	2017
4	10_P0164B	SRR9971102	South Africa	PRAXIS (this study)	Southern Africa	27	11	2017
4	10_P0191B	SRR9971344	South Africa	PRAXIS (this study)	Southern Africa	8	2	2018
4	10_R0012B	SRR9971103	South Africa	Other AHRI KZN study	Southern Africa	24	2	2017
4	10_R0027B	SRR9971104	South Africa	Other AHRI KZN study	Southern Africa	31	3	2017
4	10_R0096E	SRR9971379	South Africa	Other AHRI KZN study	Southern Africa	3	11	2017
4	11_H0010B	SRR9971273	South Africa	Other AHRI KZN study	Southern Africa	3	3	2016
4	11_H0031B	SRR9971278	South Africa	Other AHRI KZN study	Southern Africa	29	8	2016
4	11_H0120B	SRR9971246	South Africa	Other AHRI KZN study	Southern Africa	15	1	2018
4	11_P0173B	SRR9971378	South Africa	PRAXIS (this study)	Southern Africa	20	12	2017
4	11_P0190B	SRR9971070	South Africa	PRAXIS (this study)	Southern Africa	7	2	2018
4	11_R0021B	SRR9970985	South Africa	Other AHRI KZN study	Southern Africa	17	3	2017
4	12_H0131B	SRR9971178	South Africa	Other AHRI KZN study	Southern Africa	7	3	2018
4	12_H0137B	SRR9971177	South Africa	Other AHRI KZN study	Southern Africa	16	4	2018
4	12_H0145B	SRR9971174	South Africa	Other AHRI KZN study	Southern Africa	31	5	2018
4	12_H0146B	SRR9971173	South Africa	Other AHRI KZN study	Southern Africa	31	5	2018
4	12_H0147B	SRR9971176	South Africa	Other AHRI KZN study	Southern Africa	31	5	2018
4	12_H0148B	SRR9971175	South Africa	Other AHRI KZN study	Southern Africa	6	6	2018
4	12_H0153B	SRR9971170	South Africa	Other AHRI KZN study	Southern Africa	26	6	2018
4	12_H0154B	SRR9971169	South Africa	Other AHRI KZN study	Southern Africa	5	7	2018
4	12_K0011B	SRR9971172	South Africa	Other AHRI KZN study	Southern Africa	4	1	2018
4	12_P3002B	SRR9971171	South Africa	PRAXIS (this study)	Southern Africa	16	5	2018
4	12_P3409B	SRR9971233	South Africa	PRAXIS (this study)	Southern Africa	27	6	2018
4	12_R0014B	SRR9970984	South Africa	Other AHRI KZN study	Southern Africa	6	3	2017
4	13_H0142B	SRR9971234	South Africa	Other AHRI KZN study	Southern Africa	24	5	2018
4	13_K0041B	SRR11349174	South Africa	Other AHRI KZN study	Southern Africa	11	4	2018
4	13_K0054B	SRR9971232	South Africa	Other AHRI KZN study	Southern Africa	31	5	2018
4	13_K0058B	SRR9971229	South Africa	Other AHRI KZN study	Southern Africa	15	6	2018
4	13_K0059B	SRR9971230	South Africa	Other AHRI KZN study	Southern Africa	19	6	2018
4	13_P3503B	SRR9971227	South Africa	PRAXIS (this study)	Southern Africa	18	5	2018
4	13_P3505B	SRR9971228	South Africa	PRAXIS (this study)	Southern Africa	4	6	2018
4	14_H0158B	SRR9971225	South Africa	Other AHRI KZN study	Southern Africa	27	7	2018
4	14_H0160B	SRR9971226	South Africa	Other AHRI KZN study	Southern Africa	8	8	2018
4	14_K0049B	SRR9971096	South Africa	Other AHRI KZN study	Southern Africa	18	5	2018
4	14_P3016B	SRR9971095	South Africa	PRAXIS (this study)	Southern Africa	3	8	2018
4	15_H0149B	SRR9971090	South Africa	Other AHRI KZN study	Southern Africa	12	6	2018
4	15_P3507B	SRR9971153	South Africa	PRAXIS (this study)	Southern Africa	6	6	2018
4	16_P0001B	SRR9970983	South Africa	PRAXIS (this study)	Southern Africa	17	1	2017
4	16_R0004B	SRR11349157	South Africa	Other AHRI KZN study	Southern Africa	1	2	2017
4	16_R0024B	SRR9970980	South Africa	Other AHRI KZN study	Southern Africa	24	3	2017
4	16_R0029B	SRR9970979	South Africa	Other AHRI KZN study	Southern Africa	4	4	2017
4	16_R0031B	SRR9970978	South Africa	Other AHRI KZN study	Southern Africa	11	4	2017
4	16_R0039B	SRR9971026	South Africa	Other AHRI KZN study	Southern Africa	28	4	2017
4	21_H0164B	SRR9971152	South Africa	Other AHRI KZN study	Southern Africa	24	8	2018
4	21_H0165B	SRR9971151	South Africa	Other AHRI KZN study	Southern Africa	29	8	2018
4	21_H0167B	SRR9971094	South Africa	Other AHRI KZN study	Southern Africa	4	9	2018
4	21_H0169B	SRR9971093	South Africa	Other AHRI KZN study	Southern Africa	20	9	2018
4	21_K0029B	SRR9971092	South Africa	Other AHRI KZN study	Southern Africa	27	2	2018
4	21_K0030B	SRR9971091	South Africa	Other AHRI KZN study	Southern Africa	1	3	2018
4	21_K0031B	SRR9971370	South Africa	Other AHRI KZN study	Southern Africa	8	3	2018
4	21_K0044B	SRR9971371	South Africa	Other AHRI KZN study	Southern Africa	8	5	2018
4	21_K0046B	SRR9971376	South Africa	Other AHRI KZN study	Southern Africa	8	5	2018

4	21_K0051B	SRR9971377	South Africa	Other AHRI KZN study	Southern Africa	22	5	2018
4	21_K0066B	SRR9971282	South Africa	Other AHRI KZN study	Southern Africa	24	7	2018
4	21_K0073B	SRR9971283	South Africa	Other AHRI KZN study	Southern Africa	29	8	2018
4	21_P3021B	SRR9971067	South Africa	PRAXIS (this study)	Southern Africa	3	9	2018
4	21_P3028B	SRR9971373	South Africa	PRAXIS (this study)	Southern Africa	4	10	2018
4	21_P3516B	SRR9971374	South Africa	PRAXIS (this study)	Southern Africa	10	8	2018
4	21_P3519B	SRR9971375	South Africa	PRAXIS (this study)	Southern Africa	16	8	2018
4	21_P3521B	SRR9971281	South Africa	PRAXIS (this study)	Southern Africa	29	8	2018
4	21_P3532B	SRR9971275	South Africa	PRAXIS (this study)	Southern Africa	4	10	2018
4	22_H0174B	SRR9971348	South Africa	Other AHRI KZN study	Southern Africa	23	10	2018
4	22_K0043B	SRR9971347	South Africa	Other AHRI KZN study	Southern Africa	24	4	2018
4	22_K0047B	SRR9971049	South Africa	Other AHRI KZN study	Southern Africa	11	5	2018
4	22_K0075B	SRR9971048	South Africa	Other AHRI KZN study	Southern Africa	7	9	2018
4	22_K0076B	SRR9971051	South Africa	Other AHRI KZN study	Southern Africa	17	9	2018
4	22_P3026B	SRR9971050	South Africa	PRAXIS (this study)	Southern Africa	17	9	2018
4	22_P3027B	SRR9971053	South Africa	PRAXIS (this study)	Southern Africa	27	9	2018
4	22_P3032B	SRR9971052	South Africa	PRAXIS (this study)	Southern Africa	25	10	2018
4	22_P3417B	SRR9971331	South Africa	PRAXIS (this study)	Southern Africa	17	9	2018
4	22_P3527B	SRR9971332	South Africa	PRAXIS (this study)	Southern Africa	13	9	2018
4	22_P3529B	SRR9971335	South Africa	PRAXIS (this study)	Southern Africa	27	9	2018
4	22_P3530B	SRR9971336	South Africa	PRAXIS (this study)	Southern Africa	3	10	2018
4	23_H0176B	SRR9971333	South Africa	Other AHRI KZN study	Southern Africa	8	11	2018
4	23_H0181B	SRR9971334	South Africa	Other AHRI KZN study	Southern Africa	22	11	2018
4	23_H0182B	SRR9971339	South Africa	Other AHRI KZN study	Southern Africa	29	11	2018
4	23_H0187B	SRR9971340	South Africa	Other AHRI KZN study	Southern Africa	12	6	2018
4	23_K0015B	SRR9971337	South Africa	Other AHRI KZN study	Southern Africa	11	1	2018
4	23_K0080B	SRR9971338	South Africa	Other AHRI KZN study	Southern Africa	2	10	2018
4	23_K0091B	SRR9971123	South Africa	Other AHRI KZN study	Southern Africa	6	11	2018
4	23_K0093B	SRR9971122	South Africa	Other AHRI KZN study	Southern Africa	9	11	2018
4	23_K0094B	SRR9970993	South Africa	Other AHRI KZN study	Southern Africa	14	11	2018
4	23_K0095B	SRR9970992	South Africa	Other AHRI KZN study	Southern Africa	19	11	2018
4	23_K0098B	SRR9970991	South Africa	Other AHRI KZN study	Southern Africa	22	11	2018
4	23_K0101B	SRR9971165	South Africa	Other AHRI KZN study	Southern Africa	17	1	2019
4	23_KF091B	SRR9970990	South Africa	Other AHRI KZN study	Southern Africa	6	11	2018
4	23_KG091B	SRR9970989	South Africa	Other AHRI KZN study	Southern Africa	6	11	2018
4	23_P3424B	SRR9970988	South Africa	PRAXIS (this study)	Southern Africa	16	11	2018
4	23_P3425B	SRR9971164	South Africa	PRAXIS (this study)	Southern Africa	22	1	2019
4	23_P3535B	SRR9970987	South Africa	PRAXIS (this study)	Southern Africa	23	10	2018
4	24_K0081B	SRR9970986	South Africa	Other AHRI KZN study	Southern Africa	4	10	2018
4	24_K0082B	SRR9971086	South Africa	Other AHRI KZN study	Southern Africa	8	10	2018
4	24_K0086B	SRR9971084	South Africa	Other AHRI KZN study	Southern Africa	19	10	2018
4	24_K0088B	SRR9971068	South Africa	Other AHRI KZN study	Southern Africa	25	10	2018
4	24_K0089B	SRR9971066	South Africa	Other AHRI KZN study	Southern Africa	26	10	2018
4	24_K0097B	SRR9971063	South Africa	Other AHRI KZN study	Southern Africa	22	11	2018
4	24_K0100B	SRR9971163	South Africa	Other AHRI KZN study	Southern Africa	17	1	2019
4	24_P3038B	SRR9971159	South Africa	PRAXIS (this study)	Southern Africa	3	12	2018
4	25_P35056	SRR9971166	South Africa	PRAXIS (this study)	Southern Africa	16	1	2019
4	ERR1633775	ERR1633775	South Africa	Publicly available	Southern Africa	1	7	2010
4	ERR1633776	ERR1633776	South Africa	Publicly available	Southern Africa	1	7	2010
4	ERR1633778	ERR1633778	South Africa	Publicly available	Southern Africa	1	7	2010
4	ERR1633779	ERR1633779	South Africa	Publicly available	Southern Africa	1	7	2010
4	ERR1633780	ERR1633780	South Africa	Publicly available	Southern Africa	1	7	2010
4	ERR1633784	ERR1633784	South Africa	Publicly available	Southern Africa	1	7	2010
4	ERR1633785	ERR1633785	South Africa	Publicly available	Southern Africa	1	7	2010
4	ERR1633786	ERR1633786	South Africa	Publicly available	Southern Africa	1	7	2010
4	ERR1633788	ERR1633788	South Africa	Publicly available	Southern Africa	1	7	2010
4	ERR1633790	ERR1633790	South Africa	Publicly available	Southern Africa	1	7	2010
4	ERR1633794	ERR1633794	South Africa	Publicly available	Southern Africa	1	7	2010
4	ERR1633795	ERR1633795	South Africa	Publicly available	Southern Africa	1	7	2010
4	ERR1633798	ERR1633798	South Africa	Publicly available	Southern Africa	1	7	2010
4	ERR1633801	ERR1633801	South Africa	Publicly available	Southern Africa	1	7	2010

4	ERR1633802	ERR1633802	South Africa	Publicly available	Southern Africa	1	7	2010
4	ERR1633805	ERR1633805	South Africa	Publicly available	Southern Africa	1	7	2010
4	ERR1633806	ERR1633806	South Africa	Publicly available	Southern Africa	1	7	2010
4	ERR1633811	ERR1633811	South Africa	Publicly available	Southern Africa	1	7	2010
4	ERR1633813	ERR1633813	South Africa	Publicly available	Southern Africa	1	7	2010
4	ERR1633817	ERR1633817	South Africa	Publicly available	Southern Africa	1	7	2010
4	ERR1633818	ERR1633818	South Africa	Publicly available	Southern Africa	1	7	2010
4	ERR1633820	ERR1633820	South Africa	Publicly available	Southern Africa	1	7	2010
4	ERR1633821	ERR1633821	South Africa	Publicly available	Southern Africa	1	7	2010
4	ERR1633827	ERR1633827	South Africa	Publicly available	Southern Africa	1	7	2010
4	ERR1633830	ERR1633830	South Africa	Publicly available	Southern Africa	1	7	2010
4	ERR1633832	ERR1633832	South Africa	Publicly available	Southern Africa	1	7	2010
4	ERR1633833	ERR1633833	South Africa	Publicly available	Southern Africa	1	7	2010
4	ERR1633837	ERR1633837	South Africa	Publicly available	Southern Africa	1	7	2010
4	ERR1633838	ERR1633838	South Africa	Publicly available	Southern Africa	1	7	2010
4	ERR1633839	ERR1633839	South Africa	Publicly available	Southern Africa	1	7	2010
4	ERR1633841	ERR1633841	South Africa	Publicly available	Southern Africa	1	7	2010
4	ERR1633845	ERR1633845	South Africa	Publicly available	Southern Africa	1	7	2010
4	ERR1633846	ERR1633846	South Africa	Publicly available	Southern Africa	1	7	2010
4	ERR1633847	ERR1633847	South Africa	Publicly available	Southern Africa	1	7	2010
4	ERR1633851	ERR1633851	South Africa	Publicly available	Southern Africa	1	7	2010
4	ERR1633854	ERR1633854	South Africa	Publicly available	Southern Africa	1	7	2010
4	ERR1633855	ERR1633855	South Africa	Publicly available	Southern Africa	1	7	2010
4	ERR1633856	ERR1633856	South Africa	Publicly available	Southern Africa	1	7	2010
4	ERR1633858	ERR1633858	South Africa	Publicly available	Southern Africa	1	7	2010
4	ERR1633865	ERR1633865	South Africa	Publicly available	Southern Africa	1	7	2010
4	ERR1633867	ERR1633867	South Africa	Publicly available	Southern Africa	1	7	2010
4	ERR1633870	ERR1633870	South Africa	Publicly available	Southern Africa	1	7	2010
4	ERR1633871	ERR1633871	South Africa	Publicly available	Southern Africa	1	7	2010
4	ERR1633874	ERR1633874	South Africa	Publicly available	Southern Africa	1	7	2010
4	ERR1633875	ERR1633875	South Africa	Publicly available	Southern Africa	1	7	2010
4	ERR1633876	ERR1633876	South Africa	Publicly available	Southern Africa	1	7	2010
4	ERR1633877	ERR1633877	South Africa	Publicly available	Southern Africa	1	7	2010
4	ERR1633879	ERR1633879	South Africa	Publicly available	Southern Africa	1	7	2010
4	ERR1633883	ERR1633883	South Africa	Publicly available	Southern Africa	1	7	2010
4	ERR1633884	ERR1633884	South Africa	Publicly available	Southern Africa	1	7	2010
4	ERR1633891	ERR1633891	South Africa	Publicly available	Southern Africa	1	7	2010
4	ERR1633894	ERR1633894	South Africa	Publicly available	Southern Africa	1	7	2010
4	ERR1633897	ERR1633897	South Africa	Publicly available	Southern Africa	1	7	2010
4	ERR1633898	ERR1633898	South Africa	Publicly available	Southern Africa	1	7	2010
4	ERR1633899	ERR1633899	South Africa	Publicly available	Southern Africa	1	7	2010
4	ERR1633900	ERR1633900	South Africa	Publicly available	Southern Africa	1	7	2010
4	ERR1633902	ERR1633902	South Africa	Publicly available	Southern Africa	1	7	2010
4	ERR1633903	ERR1633903	South Africa	Publicly available	Southern Africa	1	7	2010
4	ERR1633904	ERR1633904	South Africa	Publicly available	Southern Africa	1	7	2010
4	ERR1633905	ERR1633905	South Africa	Publicly available	Southern Africa	1	7	2010
4	ERR1633906	ERR1633906	South Africa	Publicly available	Southern Africa	1	7	2010
4	ERR1633908	ERR1633908	South Africa	Publicly available	Southern Africa	1	7	2010
4	ERR1633914	ERR1633914	South Africa	Publicly available	Southern Africa	1	7	2010
4	ERR1633917	ERR1633917	South Africa	Publicly available	Southern Africa	1	7	2010
4	ERR1633918	ERR1633918	South Africa	Publicly available	Southern Africa	1	7	2010
4	ERR1633921	ERR1633921	South Africa	Publicly available	Southern Africa	1	7	2010
4	ERR1633923	ERR1633923	South Africa	Publicly available	Southern Africa	1	7	2010
4	ERR1633924	ERR1633924	South Africa	Publicly available	Southern Africa	1	7	2010
4	ERR1633929	ERR1633929	South Africa	Publicly available	Southern Africa	1	7	2010
4	ERR1633930	ERR1633930	South Africa	Publicly available	Southern Africa	1	7	2010
4	ERR1633934	ERR1633934	South Africa	Publicly available	Southern Africa	1	7	2010
4	ERR1633936	ERR1633936	South Africa	Publicly available	Southern Africa	1	7	2010
4	ERR1633940	ERR1633940	South Africa	Publicly available	Southern Africa	1	7	2010
4	ERR1633942	ERR1633942	South Africa	Publicly available	Southern Africa	1	7	2010
4	ERR1633947	ERR1633947	South Africa	Publicly available	Southern Africa	1	7	2010

4	ERR1633952	ERR1633952	South Africa	Publicly available	Southern Africa	1	7	2010
4	ERR1633953	ERR1633953	South Africa	Publicly available	Southern Africa	1	7	2010
4	ERR1633954	ERR1633954	South Africa	Publicly available	Southern Africa	1	7	2010
4	ERR1633957	ERR1633957	South Africa	Publicly available	Southern Africa	1	7	2010
4	ERR1633959	ERR1633959	South Africa	Publicly available	Southern Africa	1	7	2010
4	ERR1633963	ERR1633963	South Africa	Publicly available	Southern Africa	1	7	2010
4	ERR1873391	ERR1873391	South Africa	Publicly available	Southern Africa	1	7	2010
4	ERR1873392	ERR1873392	South Africa	Publicly available	Southern Africa	1	7	2010
4	ERR1873400	ERR1873400	South Africa	Publicly available	Southern Africa	1	7	2010
4	ERR1873405	ERR1873405	South Africa	Publicly available	Southern Africa	1	7	2010
4	ERR1873433	ERR1873433	South Africa	Publicly available	Southern Africa	1	7	2010
4	ERR1873435	ERR1873435	South Africa	Publicly available	Southern Africa	1	7	2010
4	ERR1873441	ERR1873441	South Africa	Publicly available	Southern Africa	1	7	2010
4	ERR1873444	ERR1873444	South Africa	Publicly available	Southern Africa	1	7	2010
4	ERR1873446	ERR1873446	South Africa	Publicly available	Southern Africa	1	7	2010
4	ERR1873448	ERR1873448	South Africa	Publicly available	Southern Africa	1	7	2010
4	ERR1873449	ERR1873449	South Africa	Publicly available	Southern Africa	1	7	2010
4	ERR1873459	ERR1873459	South Africa	Publicly available	Southern Africa	1	7	2010
4	ERR1873462	ERR1873462	South Africa	Publicly available	Southern Africa	1	7	2011
4	ERR1873475	ERR1873475	South Africa	Publicly available	Southern Africa	1	7	2011
4	ERR1873483	ERR1873483	South Africa	Publicly available	Southern Africa	1	7	2009
4	ERR1873506	ERR1873506	South Africa	Publicly available	Southern Africa	1	7	2009
4	ERR1873511	ERR1873511	South Africa	Publicly available	Southern Africa	1	7	2009
4	ERR1873512	ERR1873512	South Africa	Publicly available	Southern Africa	1	7	2010
4	ERR1873517	ERR1873517	South Africa	Publicly available	Southern Africa	1	7	2011
4	ERR1873518	ERR1873518	South Africa	Publicly available	Southern Africa	1	7	2010
4	ERR1873531	ERR1873531	South Africa	Publicly available	Southern Africa	1	7	2010
4	ERR1873534	ERR1873534	South Africa	Publicly available	Southern Africa	1	7	2011
4	ERR1873540	ERR1873540	South Africa	Publicly available	Southern Africa	1	7	2010
4	ERR1873546	ERR1873546	South Africa	Publicly available	Southern Africa	1	7	2010
4	ERR1873550	ERR1873550	South Africa	Publicly available	Southern Africa	1	7	2010
4	ERR1873554	ERR1873554	South Africa	Publicly available	Southern Africa	1	7	2010
4	ERR245649	ERR245649	Malawi	Publicly available	Southern Africa	1	7	2004
4	ERR245650	ERR245650	Malawi	Publicly available	Southern Africa	1	7	2004
4	ERR245653	ERR245653	Malawi	Publicly available	Southern Africa	1	7	2004
4	ERR245658	ERR245658	Malawi	Publicly available	Southern Africa	1	7	2004
4	ERR245659	ERR245659	Malawi	Publicly available	Southern Africa	1	7	2004
4	ERR245661	ERR245661	Malawi	Publicly available	Southern Africa	1	7	2005
4	ERR245664	ERR245664	Malawi	Publicly available	Southern Africa	1	7	2005
4	ERR245665	ERR245665	Malawi	Publicly available	Southern Africa	1	7	1999
4	ERR245666	ERR245666	Malawi	Publicly available	Southern Africa	1	7	1999
4	ERR245667	ERR245667	Malawi	Publicly available	Southern Africa	1	7	2001
4	ERR245673	ERR245673	Malawi	Publicly available	Southern Africa	1	7	2005
4	ERR245681	ERR245681	Malawi	Publicly available	Southern Africa	1	7	2005
4	ERR245683	ERR245683	Malawi	Publicly available	Southern Africa	1	7	2006
4	ERR245685	ERR245685	Malawi	Publicly available	Southern Africa	1	7	2006
4	ERR245686	ERR245686	Malawi	Publicly available	Southern Africa	1	7	2006
4	ERR245690	ERR245690	Malawi	Publicly available	Southern Africa	1	7	2006
4	ERR245693	ERR245693	Malawi	Publicly available	Southern Africa	1	7	2006
4	ERR245697	ERR245697	Malawi	Publicly available	Southern Africa	1	7	2006
4	ERR245700	ERR245700	Malawi	Publicly available	Southern Africa	1	7	2005
4	ERR245701	ERR245701	Malawi	Publicly available	Southern Africa	1	7	2005
4	ERR245706	ERR245706	Malawi	Publicly available	Southern Africa	1	7	2006
4	ERR245707	ERR245707	Malawi	Publicly available	Southern Africa	1	7	2005
4	ERR245708	ERR245708	Malawi	Publicly available	Southern Africa	1	7	2006
4	ERR245709	ERR245709	Malawi	Publicly available	Southern Africa	1	7	2006
4	ERR245711	ERR245711	Malawi	Publicly available	Southern Africa	1	7	2006
4	ERR245712	ERR245712	Malawi	Publicly available	Southern Africa	1	7	2006
4	ERR245714	ERR245714	Malawi	Publicly available	Southern Africa	1	7	2006
4	ERR245718	ERR245718	Malawi	Publicly available	Southern Africa	1	7	2005
4	ERR245719	ERR245719	Malawi	Publicly available	Southern Africa	1	7	2007

4	ERR245724	ERR245724	Malawi	Publicly available	Southern Africa	1	7	2005
4	ERR245725	ERR245725	Malawi	Publicly available	Southern Africa	1	7	2007
4	ERR245729	ERR245729	Malawi	Publicly available	Southern Africa	1	7	2007
4	ERR245730	ERR245730	Malawi	Publicly available	Southern Africa	1	7	2007
4	ERR245731	ERR245731	Malawi	Publicly available	Southern Africa	1	7	2007
4	ERR245733	ERR245733	Malawi	Publicly available	Southern Africa	1	7	2007
4	ERR245736	ERR245736	Malawi	Publicly available	Southern Africa	1	7	2007
4	ERR245737	ERR245737	Malawi	Publicly available	Southern Africa	1	7	2007
4	ERR245738	ERR245738	Malawi	Publicly available	Southern Africa	1	7	2007
4	ERR245740	ERR245740	Malawi	Publicly available	Southern Africa	1	7	2007
4	ERR245741	ERR245741	Malawi	Publicly available	Southern Africa	1	7	2008
4	ERR245743	ERR245743	Malawi	Publicly available	Southern Africa	1	7	1997
4	ERR245744	ERR245744	Malawi	Publicly available	Southern Africa	1	7	1997
4	ERR245745	ERR245745	Malawi	Publicly available	Southern Africa	1	7	1997
4	ERR245746	ERR245746	Malawi	Publicly available	Southern Africa	1	7	1997
4	ERR245749	ERR245749	Malawi	Publicly available	Southern Africa	1	7	1999
4	ERR245750	ERR245750	Malawi	Publicly available	Southern Africa	1	7	1999
4	ERR245752	ERR245752	Malawi	Publicly available	Southern Africa	1	7	2000
4	ERR245755	ERR245755	Malawi	Publicly available	Southern Africa	1	7	2000
4	ERR245756	ERR245756	Malawi	Publicly available	Southern Africa	1	7	2000
4	ERR245758	ERR245758	Malawi	Publicly available	Southern Africa	1	7	2000
4	ERR245759	ERR245759	Malawi	Publicly available	Southern Africa	1	7	2000
4	ERR550741	ERR550741	eSwatini	Publicly available	Southern Africa	1	7	2011
4	ERR550861	ERR550861	eSwatini	Publicly available	Southern Africa	1	7	2011
4	ERR551014	ERR551014	eSwatini	Publicly available	Southern Africa	1	7	2012
4	ERR551280	ERR551280	eSwatini	Publicly available	Southern Africa	1	7	2012
4	ERR551536	ERR551536	eSwatini	Publicly available	Southern Africa	1	7	2012
4	ERR551969	ERR551969	eSwatini	Publicly available	Southern Africa	1	7	2009
4	ERR552070	ERR552070	eSwatini	Publicly available	Southern Africa	1	7	2012
4	ERR552124	ERR552124	eSwatini	Publicly available	Southern Africa	1	7	2012
4	ERR552150	ERR552150	eSwatini	Publicly available	Southern Africa	1	7	2009
4	ERR552193	ERR552193	eSwatini	Publicly available	Southern Africa	1	7	2009
4	ERR552405	ERR552405	eSwatini	Publicly available	Southern Africa	1	7	2009
4	ERR552484	ERR552484	eSwatini	Publicly available	Southern Africa	1	7	2009
4	ERR552491	ERR552491	eSwatini	Publicly available	Southern Africa	1	7	2010
4	ERR552496	ERR552496	eSwatini	Publicly available	Southern Africa	1	7	2009
4	ERR552540	ERR552540	eSwatini	Publicly available	Southern Africa	1	7	2009
4	ERR552572	ERR552572	eSwatini	Publicly available	Southern Africa	1	7	2009
4	ERR552612	ERR552612	eSwatini	Publicly available	Southern Africa	1	7	2009
4	ERR552745	ERR552745	eSwatini	Publicly available	Southern Africa	1	7	2009
4	ERR552754	ERR552754	eSwatini	Publicly available	Southern Africa	1	7	2010
4	ERR552764	ERR552764	eSwatini	Publicly available	Southern Africa	1	7	2009
4	ERR553221	ERR553221	eSwatini	Publicly available	Southern Africa	1	7	2009
4	ERR553230	ERR553230	eSwatini	Publicly available	Southern Africa	1	7	2009
4	ERR553242	ERR553242	eSwatini	Publicly available	Southern Africa	1	7	2009
4	ERR553357	ERR553357	eSwatini	Publicly available	Southern Africa	1	7	2010
4	KR_F1002B	SRR9971296	South Africa	Publicly available	Southern Africa	22	6	2015
4	KR_F1013B	SRR11349134	South Africa	Publicly available	Southern Africa	17	8	2015
4	KR_F1016B	SRR11349133	South Africa	Publicly available	Southern Africa	17	8	2015
4	KR_F1019B	SRR9971312	South Africa	Publicly available	Southern Africa	24	8	2015
4	KR_F1053B	SRR9971014	South Africa	Publicly available	Southern Africa	9	11	2015
4	KR_F1057B	SRR9971003	South Africa	Publicly available	Southern Africa	16	11	2015
4	KR_F1062B	SRR11349206	South Africa	Publicly available	Southern Africa	30	11	2015
4	KR_F1070B	SRR11349203	South Africa	Publicly available	Southern Africa	21	12	2015
4	KR_F1074B	SRR11349202	South Africa	Publicly available	Southern Africa	11	1	2016
4	KR_F1075B	SRR9971276	South Africa	Publicly available	Southern Africa	11	1	2016
4	KR_F1077B	SRR11349201	South Africa	Publicly available	Southern Africa	13	1	2016
4	KR_F1079B	SRR11349200	South Africa	Publicly available	Southern Africa	18	1	2016
4	KR_F1080B	SRR9971372	South Africa	Publicly available	Southern Africa	18	1	2016
4	KR_F1085B	SRR9971280	South Africa	Publicly available	Southern Africa	27	1	2016
4	KR_F1094B	SRR11349199	South Africa	Publicly available	Southern Africa	10	2	2016

4	KR_F1096B	SRR9971047	South Africa	Publicly available	Southern Africa	22	2	2016
4	KR_F1107B	SRR9971046	South Africa	Publicly available	Southern Africa	9	3	2016
4	KR_F1123B	SRR11349198	South Africa	Publicly available	Southern Africa	23	5	2016
4	SRR1011465	SRR1011465	South Africa	Publicly available	Southern Africa	1	7	2013
4	SRR1047971	SRR1047971	South Africa	Publicly available	Southern Africa	1	7	1995
4	SRR1140899	SRR1140899	South Africa	Publicly available	Southern Africa	22	5	2013
4	SRR1140924	SRR1140924	South Africa	Publicly available	Southern Africa	15	4	2013
4	SRR1140926	SRR1140926	South Africa	Publicly available	Southern Africa	4	4	2013
4	SRR1140927	SRR1140927	South Africa	Publicly available	Southern Africa	30	1	2013
4	SRR1140929	SRR1140929	South Africa	Publicly available	Southern Africa	8	4	2013
4	SRR1140930	SRR1140930	South Africa	Publicly available	Southern Africa	10	5	2013
4	SRR1140932	SRR1140932	South Africa	Publicly available	Southern Africa	31	5	2013
4	SRR1140934	SRR1140934	South Africa	Publicly available	Southern Africa	22	5	2013
4	SRR1140936	SRR1140936	South Africa	Publicly available	Southern Africa	14	6	2013
4	SRR1140938	SRR1140938	South Africa	Publicly available	Southern Africa	10	4	2013
4	SRR1140941	SRR1140941	South Africa	Publicly available	Southern Africa	14	6	2013
4	SRR1140944	SRR1140944	South Africa	Publicly available	Southern Africa	31	5	2013
4	SRR1140945	SRR1140945	South Africa	Publicly available	Southern Africa	24	4	2013
4	SRR1140946	SRR1140946	South Africa	Publicly available	Southern Africa	27	3	2013
4	SRR1140949	SRR1140949	South Africa	Publicly available	Southern Africa	14	6	2013
4	SRR1140951	SRR1140951	South Africa	Publicly available	Southern Africa	5	4	2013
4	SRR1140952	SRR1140952	South Africa	Publicly available	Southern Africa	31	5	2013
4	SRR1140953	SRR1140953	South Africa	Publicly available	Southern Africa	27	3	2013
4	SRR1140954	SRR1140954	South Africa	Publicly available	Southern Africa	27	3	2013
4	SRR1140955	SRR1140955	South Africa	Publicly available	Southern Africa	4	4	2013
4	SRR1140957	SRR1140957	South Africa	Publicly available	Southern Africa	17	4	2013
4	SRR1140958	SRR1140958	South Africa	Publicly available	Southern Africa	4	2	2013
4	SRR1140960	SRR1140960	South Africa	Publicly available	Southern Africa	8	4	2013
4	SRR1140962	SRR1140962	South Africa	Publicly available	Southern Africa	25	3	2013
4	SRR1140965	SRR1140965	South Africa	Publicly available	Southern Africa	22	5	2013
4	SRR1140966	SRR1140966	South Africa	Publicly available	Southern Africa	6	4	2013
4	SRR1172753	SRR1172753	South Africa	Publicly available	Southern Africa	31	5	2013
4	SRR1172793	SRR1172793	South Africa	Publicly available	Southern Africa	6	8	2013
4	SRR1172836	SRR1172836	South Africa	Publicly available	Southern Africa	10	6	2013
4	SRR1172888	SRR1172888	South Africa	Publicly available	Southern Africa	13	3	2013
4	SRR1172935	SRR1172935	South Africa	Publicly available	Southern Africa	29	7	2013
4	SRR1173101	SRR1173101	South Africa	Publicly available	Southern Africa	17	5	2013
4	SRR1173119	SRR1173119	South Africa	Publicly available	Southern Africa	29	5	2013
4	SRR1173167	SRR1173167	South Africa	Publicly available	Southern Africa	12	6	2013
4	SRR1173492	SRR1173492	South Africa	Publicly available	Southern Africa	15	1	2013
4	SRR1175028	SRR1175028	South Africa	Publicly available	Southern Africa	11	12	2012
4	SRR1175301	SRR1175301	South Africa	Publicly available	Southern Africa	16	6	2013
4	SRR1175303	SRR1175303	South Africa	Publicly available	Southern Africa	23	4	2013
4	SRR1175305	SRR1175305	South Africa	Publicly available	Southern Africa	31	5	2013
4	SRR1175307	SRR1175307	South Africa	Publicly available	Southern Africa	26	7	2013
4	SRR1175329	SRR1175329	South Africa	Publicly available	Southern Africa	24	5	2013
4	SRR1175354	SRR1175354	South Africa	Publicly available	Southern Africa	23	7	2013
4	SRR1180299	SRR1180299	South Africa	Publicly available	Southern Africa	1	7	2013
4	SRR1180349	SRR1180349	South Africa	Publicly available	Southern Africa	27	8	2013
4	SRR1180362	SRR1180362	South Africa	Publicly available	Southern Africa	8	11	2012
4	SRR1180364	SRR1180364	South Africa	Publicly available	Southern Africa	15	7	2013
4	SRR1180368	SRR1180368	South Africa	Publicly available	Southern Africa	17	4	2013
4	SRR1180369	SRR1180369	South Africa	Publicly available	Southern Africa	5	8	2013
4	SRR1180371	SRR1180371	South Africa	Publicly available	Southern Africa	9	4	2013
4	SRR1180372	SRR1180372	South Africa	Publicly available	Southern Africa	30	4	2013
4	SRR1180376	SRR1180376	South Africa	Publicly available	Southern Africa	16	4	2013
4	SRR1180379	SRR1180379	South Africa	Publicly available	Southern Africa	5	8	2013
4	SRR1180388	SRR1180388	South Africa	Publicly available	Southern Africa	25	7	2013
4	SRR1180392	SRR1180392	South Africa	Publicly available	Southern Africa	29	5	2013
4	SRR1180395	SRR1180395	South Africa	Publicly available	Southern Africa	17	5	2013
4	SRR1180402	SRR1180402	South Africa	Publicly available	Southern Africa	3	4	2013

4	SRR1180403	SRR1180403	South Africa	Publicly available	Southern Africa	3	6	2013
4	SRR1180407	SRR1180407	South Africa	Publicly available	Southern Africa	30	7	2013
4	SRR1180417	SRR1180417	South Africa	Publicly available	Southern Africa	12	2	2013
4	SRR1180419	SRR1180419	South Africa	Publicly available	Southern Africa	18	7	2013
4	SRR1180422	SRR1180422	South Africa	Publicly available	Southern Africa	4	6	2013
4	SRR1180424	SRR1180424	South Africa	Publicly available	Southern Africa	15	1	2013
4	SRR1180428	SRR1180428	South Africa	Publicly available	Southern Africa	31	7	2013
4	SRR1180429	SRR1180429	South Africa	Publicly available	Southern Africa	21	5	2013
4	SRR1180433	SRR1180433	South Africa	Publicly available	Southern Africa	20	2	2013
4	SRR1180436	SRR1180436	South Africa	Publicly available	Southern Africa	19	7	2013
4	SRR1180439	SRR1180439	South Africa	Publicly available	Southern Africa	16	4	2013
4	SRR1180443	SRR1180443	South Africa	Publicly available	Southern Africa	30	1	2013
4	SRR1180445	SRR1180445	South Africa	Publicly available	Southern Africa	5	7	2013
4	SRR1182981	SRR1182981	South Africa	Publicly available	Southern Africa	9	4	2013
4	SRR1182986	SRR1182986	South Africa	Publicly available	Southern Africa	16	4	2013
4	SRR1183000	SRR1183000	South Africa	Publicly available	Southern Africa	15	1	2013
4	SRR1183001	SRR1183001	South Africa	Publicly available	Southern Africa	11	7	2013
4	SRR1183008	SRR1183008	South Africa	Publicly available	Southern Africa	11	7	2013
4	SRR1183017	SRR1183017	South Africa	Publicly available	Southern Africa	16	4	2013
4	SRR1183035	SRR1183035	South Africa	Publicly available	Southern Africa	10	5	2013
4	SRR1183042	SRR1183042	South Africa	Publicly available	Southern Africa	27	5	2013
4	SRR1183043	SRR1183043	South Africa	Publicly available	Southern Africa	23	7	2013
4	SRR1183053	SRR1183053	South Africa	Publicly available	Southern Africa	12	3	2013
4	SRR1183066	SRR1183066	South Africa	Publicly available	Southern Africa	21	8	2013
4	SRR1183073	SRR1183073	South Africa	Publicly available	Southern Africa	12	2	2013
4	SRR1183074	SRR1183074	South Africa	Publicly available	Southern Africa	20	2	2013
4	SRR1183081	SRR1183081	South Africa	Publicly available	Southern Africa	23	7	2013
4	SRR1183090	SRR1183090	South Africa	Publicly available	Southern Africa	23	5	2013
4	SRR1183101	SRR1183101	South Africa	Publicly available	Southern Africa	5	7	2013
4	SRR1183119	SRR1183119	South Africa	Publicly available	Southern Africa	13	3	2013
4	SRR1183124	SRR1183124	South Africa	Publicly available	Southern Africa	23	5	2013
4	SRR1183125	SRR1183125	South Africa	Publicly available	Southern Africa	4	7	2013
4	SRR1183131	SRR1183131	South Africa	Publicly available	Southern Africa	22	7	2013
4	SRR1183146	SRR1183146	South Africa	Publicly available	Southern Africa	18	7	2013
4	SRR1184293	SRR1184293	South Africa	Publicly available	Southern Africa	21	6	2013
4	SRR1184295	SRR1184295	South Africa	Publicly available	Southern Africa	15	7	2013
4	SRR1184297	SRR1184297	South Africa	Publicly available	Southern Africa	7	8	2013
4	SRR1184298	SRR1184298	South Africa	Publicly available	Southern Africa	12	7	2013
4	SRR1184299	SRR1184299	South Africa	Publicly available	Southern Africa	24	7	2013
4	SRR1184300	SRR1184300	South Africa	Publicly available	Southern Africa	19	8	2013
4	SRR1184301	SRR1184301	South Africa	Publicly available	Southern Africa	20	8	2013
4	SRR1184304	SRR1184304	South Africa	Publicly available	Southern Africa	31	5	2013
4	SRR1184305	SRR1184305	South Africa	Publicly available	Southern Africa	21	6	2013
4	SRR1184306	SRR1184306	South Africa	Publicly available	Southern Africa	29	7	2013
4	SRR1184309	SRR1184309	South Africa	Publicly available	Southern Africa	28	6	2013
4	SRR1184310	SRR1184310	South Africa	Publicly available	Southern Africa	18	7	2013
4	SRR1184313	SRR1184313	South Africa	Publicly available	Southern Africa	9	7	2013
4	SRR1184314	SRR1184314	South Africa	Publicly available	Southern Africa	11	7	2013
4	SRR1184315	SRR1184315	South Africa	Publicly available	Southern Africa	10	7	2013
4	SRR1184316	SRR1184316	South Africa	Publicly available	Southern Africa	7	8	2013
4	SRR1184317	SRR1184317	South Africa	Publicly available	Southern Africa	10	7	2013
4	SRR1184319	SRR1184319	South Africa	Publicly available	Southern Africa	24	7	2013
4	SRR1184320	SRR1184320	South Africa	Publicly available	Southern Africa	23	5	2013
4	SRR1184321	SRR1184321	South Africa	Publicly available	Southern Africa	31	7	2013
4	SRR1184324	SRR1184324	South Africa	Publicly available	Southern Africa	10	7	2013
4	SRR1184325	SRR1184325	South Africa	Publicly available	Southern Africa	19	6	2013
4	SRR1184326	SRR1184326	South Africa	Publicly available	Southern Africa	30	7	2013
4	SRR1184327	SRR1184327	South Africa	Publicly available	Southern Africa	8	8	2013
4	SRR1184328	SRR1184328	South Africa	Publicly available	Southern Africa	17	7	2013
4	SRR1184331	SRR1184331	South Africa	Publicly available	Southern Africa	12	7	2013
4	SRR1184333	SRR1184333	South Africa	Publicly available	Southern Africa	30	7	2013

4	SRR1184334	SRR1184334	South Africa	Publicly available	Southern Africa	23	8	2013
4	SRR1184335	SRR1184335	South Africa	Publicly available	Southern Africa	4	8	2013
4	SRR1184336	SRR1184336	South Africa	Publicly available	Southern Africa	24	7	2013
4	SRR1184337	SRR1184337	South Africa	Publicly available	Southern Africa	2	7	2013
4	SRR1184339	SRR1184339	South Africa	Publicly available	Southern Africa	10	6	2013
4	SRR1184340	SRR1184340	South Africa	Publicly available	Southern Africa	23	8	2013
4	SRR1184341	SRR1184341	South Africa	Publicly available	Southern Africa	11	7	2013
4	SRR1184342	SRR1184342	South Africa	Publicly available	Southern Africa	31	7	2013
4	SRR1184345	SRR1184345	South Africa	Publicly available	Southern Africa	19	6	2013
4	SRR1184347	SRR1184347	South Africa	Publicly available	Southern Africa	3	7	2013
4	SRR1184348	SRR1184348	South Africa	Publicly available	Southern Africa	12	6	2013
4	SRR1184351	SRR1184351	South Africa	Publicly available	Southern Africa	4	7	2013
4	SRR1184352	SRR1184352	South Africa	Publicly available	Southern Africa	7	6	2013
4	SRR1184353	SRR1184353	South Africa	Publicly available	Southern Africa	7	6	2013
4	SRR1184354	SRR1184354	South Africa	Publicly available	Southern Africa	25	7	2013
4	SRR1184355	SRR1184355	South Africa	Publicly available	Southern Africa	29	5	2013
4	SRR1184356	SRR1184356	South Africa	Publicly available	Southern Africa	19	6	2013
4	SRR1184359	SRR1184359	South Africa	Publicly available	Southern Africa	14	7	2013
4	SRR1184360	SRR1184360	South Africa	Publicly available	Southern Africa	1	8	2013
4	SRR1184361	SRR1184361	South Africa	Publicly available	Southern Africa	29	5	2013
4	SRR1184362	SRR1184362	South Africa	Publicly available	Southern Africa	15	7	2013
4	SRR1184363	SRR1184363	South Africa	Publicly available	Southern Africa	5	6	2013
4	SRR1184366	SRR1184366	South Africa	Publicly available	Southern Africa	24	7	2013
4	SRR1184367	SRR1184367	South Africa	Publicly available	Southern Africa	23	8	2013
4	SRR1184368	SRR1184368	South Africa	Publicly available	Southern Africa	26	7	2013
4	SRR1184369	SRR1184369	South Africa	Publicly available	Southern Africa	23	8	2013
4	SRR1184371	SRR1184371	South Africa	Publicly available	Southern Africa	12	6	2013
4	SRR1184372	SRR1184372	South Africa	Publicly available	Southern Africa	28	6	2013
4	SRR1184374	SRR1184374	South Africa	Publicly available	Southern Africa	17	7	2013
4	SRR1184375	SRR1184375	South Africa	Publicly available	Southern Africa	12	7	2013
4	SRR1184377	SRR1184377	South Africa	Publicly available	Southern Africa	22	7	2013
4	SRR1184378	SRR1184378	South Africa	Publicly available	Southern Africa	30	7	2013
4	SRR1184381	SRR1184381	South Africa	Publicly available	Southern Africa	22	7	2013
4	SRR1184382	SRR1184382	South Africa	Publicly available	Southern Africa	6	8	2013
4	SRR1184384	SRR1184384	South Africa	Publicly available	Southern Africa	3	7	2013
4	SRR1184385	SRR1184385	South Africa	Publicly available	Southern Africa	3	7	2013
4	SRR1184389	SRR1184389	South Africa	Publicly available	Southern Africa	31	5	2013
4	SRR2101766	SRR2101766	South Africa	Publicly available	Southern Africa	14	8	2013
4	SRR3055711	SRR3055711	South Africa	Publicly available	Southern Africa	1	7	1996
4	SRR3055712	SRR3055712	South Africa	Publicly available	Southern Africa	1	7	1994
4	SRR3055713	SRR3055713	South Africa	Publicly available	Southern Africa	1	7	2005
4	SRR3055714	SRR3055714	South Africa	Publicly available	Southern Africa	1	7	2005
4	SRR3055717	SRR3055717	South Africa	Publicly available	Southern Africa	1	7	2006
4	SRR3055718	SRR3055718	South Africa	Publicly available	Southern Africa	1	7	2005
4	SRR3055719	SRR3055719	South Africa	Publicly available	Southern Africa	1	7	2005
4	SRR6207275	SRR6207275	South Africa	Publicly available	Southern Africa	4	9	2012
4	SRR6207278	SRR6207278	South Africa	Publicly available	Southern Africa	30	8	2012
4	SRR6207280	SRR6207280	South Africa	Publicly available	Southern Africa	14	8	2012
4	SRR6207282	SRR6207282	South Africa	Publicly available	Southern Africa	29	6	2012
4	SRR6207283	SRR6207283	South Africa	Publicly available	Southern Africa	4	10	2012
4	SRR6207287	SRR6207287	South Africa	Publicly available	Southern Africa	11	9	2012
4	SRR6207289	SRR6207289	South Africa	Publicly available	Southern Africa	25	9	2012
4	SRR6207290	SRR6207290	South Africa	Publicly available	Southern Africa	21	9	2012
4	SRR6207292	SRR6207292	South Africa	Publicly available	Southern Africa	24	10	2012
4	SRR832978	SRR832978	South Africa	Publicly available	Southern Africa	19	3	2008
4	SRR832986	SRR832986	South Africa	Publicly available	Southern Africa	12	5	2008
4	SRR832988	SRR832988	South Africa	Publicly available	Southern Africa	27	11	2009
4	SRR832991	SRR832991	South Africa	Publicly available	Southern Africa	1	7	2008
4	SRR832995	SRR832995	South Africa	Publicly available	Southern Africa	25	2	2010
4	SRR832997	SRR832997	South Africa	Publicly available	Southern Africa	21	2	2008
4	SRR833011	SRR833011	South Africa	Publicly available	Southern Africa	17	7	2008

4	SRR833013	SRR833013	South Africa	Publicly available	Southern Africa	3	4	2008
4	SRR833015	SRR833015	South Africa	Publicly available	Southern Africa	23	1	2008
4	SRR833028	SRR833028	South Africa	Publicly available	Southern Africa	1	7	2008
4	SRR833031	SRR833031	South Africa	Publicly available	Southern Africa	9	6	2008
4	SRR833037	SRR833037	South Africa	Publicly available	Southern Africa	15	2	2008
4	SRR833040	SRR833040	South Africa	Publicly available	Southern Africa	28	6	2010
4	SRR833044	SRR833044	South Africa	Publicly available	Southern Africa	7	1	2010
4	SRR833046	SRR833046	South Africa	Publicly available	Southern Africa	11	8	2009
4	SRR833053	SRR833053	South Africa	Publicly available	Southern Africa	22	7	2008
4	SRR833056	SRR833056	South Africa	Publicly available	Southern Africa	13	5	2008
4	SRR833058	SRR833058	South Africa	Publicly available	Southern Africa	8	7	2010
4	SRR833059	SRR833059	South Africa	Publicly available	Southern Africa	25	3	2010
4	SRR833064	SRR833064	South Africa	Publicly available	Southern Africa	5	11	2010
4	SRR833065	SRR833065	South Africa	Publicly available	Southern Africa	14	8	2008
4	SRR833077	SRR833077	South Africa	Publicly available	Southern Africa	7	4	2010
4	SRR833078	SRR833078	South Africa	Publicly available	Southern Africa	29	3	2010
4	SRR833084	SRR833084	South Africa	Publicly available	Southern Africa	4	3	2010
4	SRR833086	SRR833086	South Africa	Publicly available	Southern Africa	1	3	2010
4	SRR833091	SRR833091	South Africa	Publicly available	Southern Africa	12	1	2010
4	SRR833097	SRR833097	South Africa	Publicly available	Southern Africa	3	2	2010
4	SRR833105	SRR833105	South Africa	Publicly available	Southern Africa	6	2	2008
4	SRR833111	SRR833111	South Africa	Publicly available	Southern Africa	8	1	2008
4	SRR833115	SRR833115	South Africa	Publicly available	Southern Africa	23	7	2010
4	SRR833117	SRR833117	South Africa	Publicly available	Southern Africa	30	7	2008
4	SRR833119	SRR833119	South Africa	Publicly available	Southern Africa	14	8	2008
4	SRR833120	SRR833120	South Africa	Publicly available	Southern Africa	14	8	2008
4	SRR833121	SRR833121	South Africa	Publicly available	Southern Africa	1	7	2008
4	SRR833122	SRR833122	South Africa	Publicly available	Southern Africa	25	11	2009
4	SRR833124	SRR833124	South Africa	Publicly available	Southern Africa	18	1	2010
4	SRR833130	SRR833130	South Africa	Publicly available	Southern Africa	31	7	2008
4	SRR833131	SRR833131	South Africa	Publicly available	Southern Africa	26	2	2008
4	SRR833134	SRR833134	South Africa	Publicly available	Southern Africa	13	1	2010
4	SRR833137	SRR833137	South Africa	Publicly available	Southern Africa	26	1	2009
4	SRR833140	SRR833140	South Africa	Publicly available	Southern Africa	15	7	2008
4	SRR833144	SRR833144	South Africa	Publicly available	Southern Africa	19	2	2008
4	SRR833146	SRR833146	South Africa	Publicly available	Southern Africa	20	2	2008
4	SRR833149	SRR833149	South Africa	Publicly available	Southern Africa	16	1	2008
4	SRR833150	SRR833150	South Africa	Publicly available	Southern Africa	12	5	2008
4	SRR833151	SRR833151	South Africa	Publicly available	Southern Africa	30	11	2009
4	SRR833163	SRR833163	South Africa	Publicly available	Southern Africa	25	2	2008
4	SRR833164	SRR833164	South Africa	Publicly available	Southern Africa	19	2	2008
4	SRR833173	SRR833173	South Africa	Publicly available	Southern Africa	25	3	2010
4	SRR833174	SRR833174	South Africa	Publicly available	Southern Africa	13	5	2008
4	SRR833175	SRR833175	South Africa	Publicly available	Southern Africa	2	4	2008
4	SRR833177	SRR833177	South Africa	Publicly available	Southern Africa	26	2	2010
4	SRR833179	SRR833179	South Africa	Publicly available	Southern Africa	6	1	2010
4	SRR833184	SRR833184	South Africa	Publicly available	Southern Africa	23	4	2009
4	SRR833188	SRR833188	South Africa	Publicly available	Southern Africa	21	2	2008
4	SRR833191	SRR833191	South Africa	Publicly available	Southern Africa	25	6	2010
4	SRR833194	SRR833194	South Africa	Publicly available	Southern Africa	21	5	2009
4	SRR833196	SRR833196	South Africa	Publicly available	Southern Africa	23	2	2010
4	SRR847775	SRR847775	South Africa	Publicly available	Southern Africa	25	2	2011
4	SRR847776	SRR847776	South Africa	Publicly available	Southern Africa	9	9	2011
4	SRR847777	SRR847777	South Africa	Publicly available	Southern Africa	22	12	2011
4	SRR847778	SRR847778	South Africa	Publicly available	Southern Africa	21	1	2011
4	SRR847779	SRR847779	South Africa	Publicly available	Southern Africa	9	9	2011
4	SRR847780	SRR847780	South Africa	Publicly available	Southern Africa	17	1	2012
4	SRR847781	SRR847781	South Africa	Publicly available	Southern Africa	14	12	2011
4	SRR847782	SRR847782	South Africa	Publicly available	Southern Africa	22	12	2011
4	SRR847783	SRR847783	South Africa	Publicly available	Southern Africa	14	6	2011
4	SRR847784	SRR847784	South Africa	Publicly available	Southern Africa	22	12	2011

4	SRR847785	SRR847785	South Africa	Publicly available	Southern Africa	24	8	2011
4	SRR847787	SRR847787	South Africa	Publicly available	Southern Africa	15	11	2010
4	SRR847788	SRR847788	South Africa	Publicly available	Southern Africa	4	2	2011
4	SRR847789	SRR847789	South Africa	Publicly available	Southern Africa	9	9	2011
4	SRR847790	SRR847790	South Africa	Publicly available	Southern Africa	15	11	2010
4	SRR847791	SRR847791	South Africa	Publicly available	Southern Africa	14	12	2011
4	SRR847792	SRR847792	South Africa	Publicly available	Southern Africa	4	2	2011
4	SRR847793	SRR847793	South Africa	Publicly available	Southern Africa	9	9	2011
4	SRR847794	SRR847794	South Africa	Publicly available	Southern Africa	21	1	2011
4	SRR847795	SRR847795	South Africa	Publicly available	Southern Africa	22	12	2011
4	SRR847796	SRR847796	South Africa	Publicly available	Southern Africa	23	9	2010
4	SRR847797	SRR847797	South Africa	Publicly available	Southern Africa	28	1	2011
4	SRR847798	SRR847798	South Africa	Publicly available	Southern Africa	27	1	2012
4	SRR847800	SRR847800	South Africa	Publicly available	Southern Africa	5	11	2010
4	SRR847801	SRR847801	South Africa	Publicly available	Southern Africa	24	8	2011
4	SRR847802	SRR847802	South Africa	Publicly available	Southern Africa	14	12	2011
4	SRR847803	SRR847803	South Africa	Publicly available	Southern Africa	15	11	2010
4	SRR924700	SRR924700	South Africa	Publicly available	Southern Africa	1	7	2008
4	SRR924701	SRR924701	South Africa	Publicly available	Southern Africa	1	7	2008
4	SRR924707	SRR924707	South Africa	Publicly available	Southern Africa	1	7	2008
4	SRR958195	SRR958195	South Africa	Publicly available	Southern Africa	21	7	2009
4	SRR958201	SRR958201	South Africa	Publicly available	Southern Africa	10	1	2008
4	SRR958204	SRR958204	South Africa	Publicly available	Southern Africa	23	1	2008
4	SRR958207	SRR958207	South Africa	Publicly available	Southern Africa	4	2	2008
4	SRR958210	SRR958210	South Africa	Publicly available	Southern Africa	3	3	2008
4	SRR958216	SRR958216	South Africa	Publicly available	Southern Africa	6	3	2008
4	SRR958221	SRR958221	South Africa	Publicly available	Southern Africa	3	3	2008
4	SRR958224	SRR958224	South Africa	Publicly available	Southern Africa	4	3	2008
4	SRR958225	SRR958225	South Africa	Publicly available	Southern Africa	20	3	2008

Additional References

1. Coll F, McNerney R, Guerra-Assunção JA, Glynn JR, Perdigão J, Viveiros M, et al. A robust SNP barcode for typing Mycobacterium tuberculosis complex strains. Nat Commun. 2014;5.
2. Brynildsrud OB, Pepperell CS, Suffys P, Grandjean L, Monteserin J, Debech N, et al. Global expansion of Mycobacterium tuberculosis lineage 4 shaped by colonial migration and local adaptation. Sci Adv. 2018;4(10).
3. Somoskovi A, Bruderer V, Hömke R, Bloemberg G V, Böttger EC. A mutation associated with clofazimine and bedaquiline cross-resistance in MDR-TB following bedaquiline treatment. Eur Respir J. 2015 Feb 1;45(2):554 LP – 557.
4. Villellas C, Coeck N, Meehan CJ, Lounis N, de Jong B, Rigouts L, et al. Unexpected high prevalence of resistance-associated Rv0678 variants in MDR-TB patients without documented prior use of clofazimine or bedaquiline. J Antimicrob Chemother. 2017;72(3):684–90.
5. Yang JS, Kim KJ, Choi H, Lee SH. Delamanid, bedaquiline, and linezolid minimum inhibitory concentration distributions and resistance-related gene mutations in multidrug-resistant and extensively drug-resistant tuberculosis in Korea. Ann Lab Med. 2018;38(6):563–8.
6. Andries K, Villellas C, Coeck N, Thys K, Gevers T, Vranckx L, et al. Acquired resistance of Mycobacterium tuberculosis to bedaquiline. PLoS One. 2014;9(7).
7. Xu J, Wang B, Hu M, Huo F, Guo S, Jing W, et al. Primary Clofazimine and Bedaquiline Resistance among Isolates from Patients with Multidrug-Resistant Tuberculosis. Antimicrob Agents Chemother. 2017;61(6):e00239-17.
8. Andres S, Merker M, Heyckendorf J, Kalsdorf B, Rumetshofer R, Indra A, et al. Bedaquiline-resistant Tuberculosis: Dark Clouds on the Horizon. Am J Respir Crit Care Med. 2020 Feb;rccm.201909-1819LE.
9. Ghodousi A, Rizvi AH, Baloch AQ, Ghafoor A, Khanzada FM, Qadir M, et al. Acquisition of Cross-Resistance to Bedaquiline and Clofazimine following Treatment for Tuberculosis in Pakistan. Antimicrob Agents Chemother. 2019;63(9).

10. Nimmo C, Millard J, Brien K, Moodley S, van Dorp L, Lutchminarain K, et al. Bedaquiline resistance in drug-resistant tuberculosis HIV co-infected patients. Eur Respir J. 2020 Feb;55(6).
